# Randomized Phase III Trial of Adjuvant Chemotherapy with S-1 after Curative Treatment in Patients with Squamous-Cell Carcinoma of the Head and Neck (ACTS-HNC)

**DOI:** 10.1371/journal.pone.0116965

**Published:** 2015-02-11

**Authors:** Kiyoaki Tsukahara, Akira Kubota, Yasuhisa Hasegawa, Hideki Takemura, Tomonori Terada, Takahide Taguchi, Kunihiko Nagahara, Hiroaki Nakatani, Kunitoshi Yoshino, Yuichiro Higaki, Shigemichi Iwae, Takeshi Beppu, Yutaka Hanamure, Kichinobu Tomita, Naoyuki Kohno, Kazuyoshi Kawabata, Masanori Fukushima, Satoshi Teramukai, Masato Fujii, ACTS-HNC group

**Affiliations:** 1 Department of Otolaryngology, Head and Neck Surgery, Tokyo Medical University Hachioji Medical Center, Hachioji, Japan; 2 Department Head and Neck Surgery, Kanagawa Cancer Center, Yokohama, Japan; 3 Department of Head and Neck Surgery, Aichi Cancer Center Hospital, Nagoya, Japan; 4 Department of Otolaryngology, Yokohama Rosai Hospital, Yokohama, Japan; 5 Department of Otolaryngology, Hyogo College of Medicine, Nishinomiya, Japan; 6 Department of Otorhinolaryngology, and Head and Neck Surgery, Yokohama City University, School of Medicine, Yokohama, Japan; 7 Department of Head and Neck Surgery, Kusatsu General Hospital, Kusatsu, Japan; 8 Department of Otolaryngology, Head and Neck Surgery, National Hospital Organization Fukuyama Medical Center, Fukuyama, Japan; 9 Department of Otolaryngology, Head and Neck Surgery, Osaka Medical Center for Cancer and Cardiovascular Diseases, Osaka, Japan; 10 Department of Head and Neck Surgery, National Hospital Organization Kyushu Cancer Center, Fukuoka, Japan; 11 Department of Head and Neck Surgery, Hyogo Cancer Center, Akashi, Japan; 12 Department of Head and Neck Surgery, Saitama Cancer Center, Saitama, Japan; 13 Department of Otolaryngology, Kagoshima City Hospital, Kagoshima, Japan; 14 Department of Otolaryngology, Akasaka Surge Clinic, Fukuoka, Japan; 15 Department of Otolaryngology, Head and Neck Surgery, Kyorin University School of Medicine, Mitaka, Japan; 16 Division of Head and Neck Surgery, Cancer Institute Hospital, Tokyo, Japan; 17 Translational Research Informatics Center, Kobe, Japan; 18 Innovative Clinical Research Center, Kanazawa University Hospital, Kanazawa, Japan; 19 Department of Otolaryngology, National Hospital Organization Tokyo Medical Center, Tokyo, Japan; University Clinic of Navarra, SPAIN

## Abstract

**Background:**

We conducted a phase III study to evaluate S-1 as compared with UFT as control in patients after curative therapy for stage III, IVA, or IVB squamous-cell carcinoma of the head and neck (SCCHN).

**Patients and Methods:**

Patients were randomly assigned to the UFT group (300 or 400 mg day^-1^ for 1 year) or the S-1 group (80, 100, or 120 mg day^-1^ for 1 year). The primary end point was disease-free survival (DFS). Secondary end points were relapse-free survival, overall survival (OS), and safety.

**Results:**

A total of 526 patients were enrolled, and 505 were eligible for analysis. The 3-year DFS rate was 60.0% in the UFT group and 64.1% in the S-1 group (HR, 0.87; 95%CI, 0.66-1.16; p = 0.34). The 3-year OS rate was 75.8% and 82.9%, respectively (HR, 0.64; 95% CI, 0.44-0.94; p = 0.022). Among grade 3 or higher adverse events, the incidences of leukopenia (5.2%), neutropenia (3.6%), thrombocytopenia (2.0%), and mucositis/stomatitis (2.4%) were significantly higher in the S-1 group.

**Conclusions:**

Although DFS did not differ significantly between the groups, OS was significantly better in the S-1 group than in the UFT group. S-1 is considered a treatment option after curative therapy for stage III, IVA, IVB SCCHN.

**Trial Registration:**

ClinicalTrials.gov NCT00336947 http://clinicaltrials.gov/show/NCT00336947

## Introduction

Stage III or IV, locally advanced, squamous-cell carcinoma of the head and neck (SCCHN) is usually treated by surgery or Concurrent chemoradiotherapy [1–3]. The National Comprehensive Cancer Network (NCCN) practice guidelines, the European Head and Neck Society—European Society for Medical Oncology—European Society for Radiotherapy and Oncology (EHNS-ESMO-ESTRO) clinical practice guidelines, and the Japanese practice guidelines in head and neck cancer (version 2013) recommend that high-risk patients with oral cancer, oropharyngeal cancer, hypopharyngeal cancer, or laryngeal cancer should receive postoperative chemoradiotherapy [4–6]. However, neck recurrence and distant metastasis can occur after postoperative chemoradiotherapy, and outcomes remain unsatisfactory. A meta-analysis did not provide convincing evidence of any benefits of adjuvant chemotherapy in patients with head and neck cancer [7]. Given the current situation, it would be meaningful to have an effective oral preparation that could be used for adjuvant chemotherapy on an outpatient basis.

Tegafur/uracil (UFT, Taiho Pharmaceutical Co., Ltd., Tokyo, Japan) and S-1 (TS-1, Taiho Pharmaceutical Co., Ltd.) are oral agents that are effective against SCCHN. UFT combines tegafur with uracil in a molar ratio of 1:4. Tegafur, an anticancer prodrug, is metabolized to 5-fluorouracil (5-FU). Then, 5-FU is rapidly degraded by dihydropyrimidine dehydrogenase (DPD) in blood. Uracil inhibits DPD and thereby maintains high concentrations of 5-FU [8,9]. S-1 is a fluoropyrimidine preparation that combines tegafur with gimeracil and oteracil potassium in a molar ratio of 1:0.4:1. Gimeracil in S-1 is a DPD inhibitor that is 180 times more potent than uracil in UFT, allowing high concentrations of 5-FU to be maintained [10]. Oteracil inhibits the phosphorylation of 5-FU in the gastrointestinal mucosa, thereby reducing gastrointestinal toxicity [11,12]. Two phase II studies of S-1 performed in Japan in patients with advanced or recurrent head and neck cancer (or both) respectively obtained response rates of 46.2% and 28.8% with acceptable toxicity [13,14].

A phase III study of S-1 in postoperative patients with gastric cancer (Adjuvant Chemotherapy Trial of TS-1 for Gastric Cancer [ACTS-GC]) showed that S-1 improves overall survival (OS) and relapse-free survival (RFS), as compared with surgery alone [15,16]. Furthermore, in pancreatic cancer, S-1 was superior to standard therapy with gemcitabine in terms of OS in an adjuvant setting [17].

As for UFT, phase III clinical study has been performed in patients with SCCHN. After curative surgery, patients were assigned to a control group, which received surgery alone, or to a UFT group to assess treatment effectiveness. Although there was no difference between the groups in OS or disease-free survival (DFS), the distant metastasis control rate was significantly higher in the UFT group [18].

The present ACTS-HNC (Adjuvant Chemotherapy with S-1 after Curative Treatment in Patients with Head and Neck Cancer) study was performed to evaluate the efficacy and safety of S-1 as compared with UFT as control treatment in patients with SCCHN.

## Patients and Methods

The protocol for this trial and supporting CONSORT checklist are available as supporting information; see S1 CONSORT Checklist and S1 Protocol.

### Study Design

The ACTS-HNC study was a prospective, multicenter, randomized, open-label, 2-arm, parallel-group phase III study. The study was sponsored by the Translational Research Informatics Center (TRI) and received financial support from Taiho Pharmaceutical Co., Ltd. The study was conducted in accordance with “Japanese Ethical Guidelines for Clinical Studies” and the Declaration of Helsinki and was approved by the ethics committee or institutional review board of each participating center (See S1 List: List of the name of committee/institutional review boards). Data were managed by an Academic Research Organization contracted by the sponsors and were analyzed by a study statistician (S.T.). An independent data and safety monitoring committee reviewed efficacy and safety data for interim monitoring. This study is registered with ClinicalTrials.gov, identification number NCT00336947. (http://clinicaltrials.gov/show/NCT00336947)

### Patients

**Fig 1:**
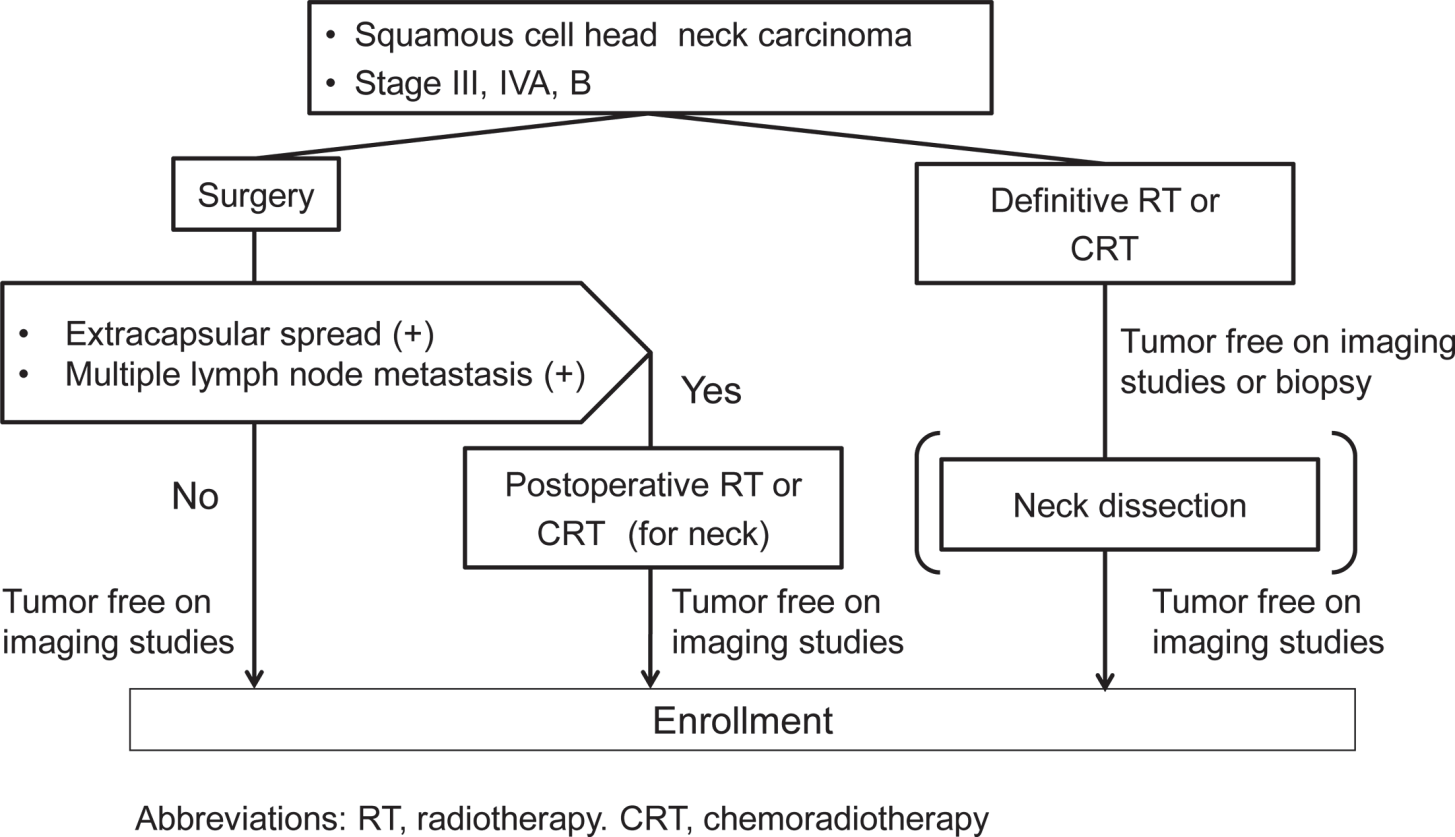
Definition of definitive treatment. Abbreviations: RT, radiotherapy. CRT, chemoradiotherapy.

Eligible patients had to have received curative surgery combined with or without postoperative chemoradiotherapy or curative radiotherapy (including chemoradiotherapy) for a diagnosis of stage III, IVA, or IVB SCCHN. No residual tumor (primary lesion or lymph nodes) was confirmed on diagnostic imaging (computed tomography, magnetic resonance imaging, positron emission tomography, ultrasonography, or chest radiography) or on biopsy (Fig. 1). The primary tumor had to be located in the maxillary sinus, oral cavity, oropharynx, hypopharynx, or larynx (excluding T3N0 tumors of the glottis). Other eligibility criteria were an age of 20 to 75 years, an Eastern Cooperative Oncology Group performance status of 0 or 1, adequate liver, renal, and bone marrow functions, no other cancer within the past 3 years, and the ability for oral intake. After the completion of curative therapy, all patients were confirmed to meet the eligibility criteria and gave written informed consent.

### Treatment

Patients were randomly assigned to treatment groups after completion of curative therapy, using four stratification factors: primary tumor site, disease stage (III, IVA, or IVB), type of curative therapy (surgery, radiotherapy, or both), and institution. In the UFT group, patients received 300 mg day^-1^ (body surface area [BSA] <1.5 m^2^) or 400 mg day^-1^ (BSA ≥1.5 m^2^) of UFT in two or three divided doses daily. In the S-1 group, patients received 80 mg day^-1^ (BSA<1.25 m^2^), 100 mg day^-1^ (BSA ≥1.25 to <1.5 m^2^), or 120 mg day^-1^ (BSA ≥1.5 m^2^) of S-1 in two divided doses daily for 2 weeks, followed by a 1-week rest [19]. The duration of treatment was 1 year in both groups.

If adverse events meeting the criteria for temporary treatment withdrawal occurred (S1 Table), treatment was discontinued and resumed when the criteria for treatment resumption (S2 Table) were satisfied. If adverse events meeting the criteria for dose reduction developed (S3 Table), the dose was reduced by one level (S4 Table and S5 Table) before resuming treatment.

### Assessments

Clinical findings, including the results of blood tests and adverse events, were evaluated on days 1, 8, and 29 after the start of treatment and at 4-week intervals from week 5 onward in the UFT group. In the S-1 group, clinical findings were evaluated on days 1 and 8 of the first course of treatment and on day 1 of each course from the second course onward. Fluoropyrimidine-related and well-known adverse events were prespecified and monitored. Adverse events were assessed according to the Common Terminology Criteria for Adverse Events (CTCAE), version 3.0. The presence or absence of recurrence and secondary cancer was confirmed every 3 to 6 months on imaging studies, pathological examination, cytologic examination, or other diagnostic techniques.

### Statistical Analysis

The primary end point was DFS, defined as the time from the date of randomization to the date of confirmation of recurrence, the diagnosis of secondary cancer, or death from any cause, whichever came first. Secondary end points were RFS, OS, and safety. RFS was defined as the time from the date of randomization to the date of confirmation of recurrence or death from any cause, whichever came first. OS was defined as the time from the date of randomization to the date of death from any cause. If no event occurred during follow-up, data on DFS, RFS, and OS were censored on the date that the patient was last confirmed to be alive. The locoregional control time was defined as the time from randomization to the diagnosis of local recurrence or cervical lymph node recurrence. The distant metastasis free time was defined as the time from randomization to the diagnosis of distant recurrence.

Assuming that the 3-year DFS rate would be 50% on the basis of past studies[20–22], with a hazard ratio (HR) of S-1 to UFT of 0.7 (3-year DFS, 61.6%), an alpha level of 0.05 (two-sided), an enrollment period of 3 years, a follow-up period of 3 years, and a statistical power of 80%, we estimated that 500 patients would be required. An interim analysis using O'Brien-Fleming boundaries was planned 1 year after the completion of enrollment[23]. The P value at the final analysis was 0.04852.

All analyses were performed on an intention-to-treat (ITT) basis in all eligible patients who received some or all of the protocol treatment. DFS, RFS, and OS curves were calculated by the Kaplan-Meier method. The treatment groups were compared with log-rank tests stratified according to the stratification factors, except for institution. To assess treatment efficacy, a Cox proportional-hazards model was used to estimate HRs and 95% confidence intervals (95% CI). Fisher’s exact test was used to evaluate differences in the incidences of grade 3 or grade 4 adverse events. All statistical analyses were done with SAS, version 9.2 (SAS Institute, Cary, NC, USA).

## Results

### Patient characteristics

**Fig 2:**
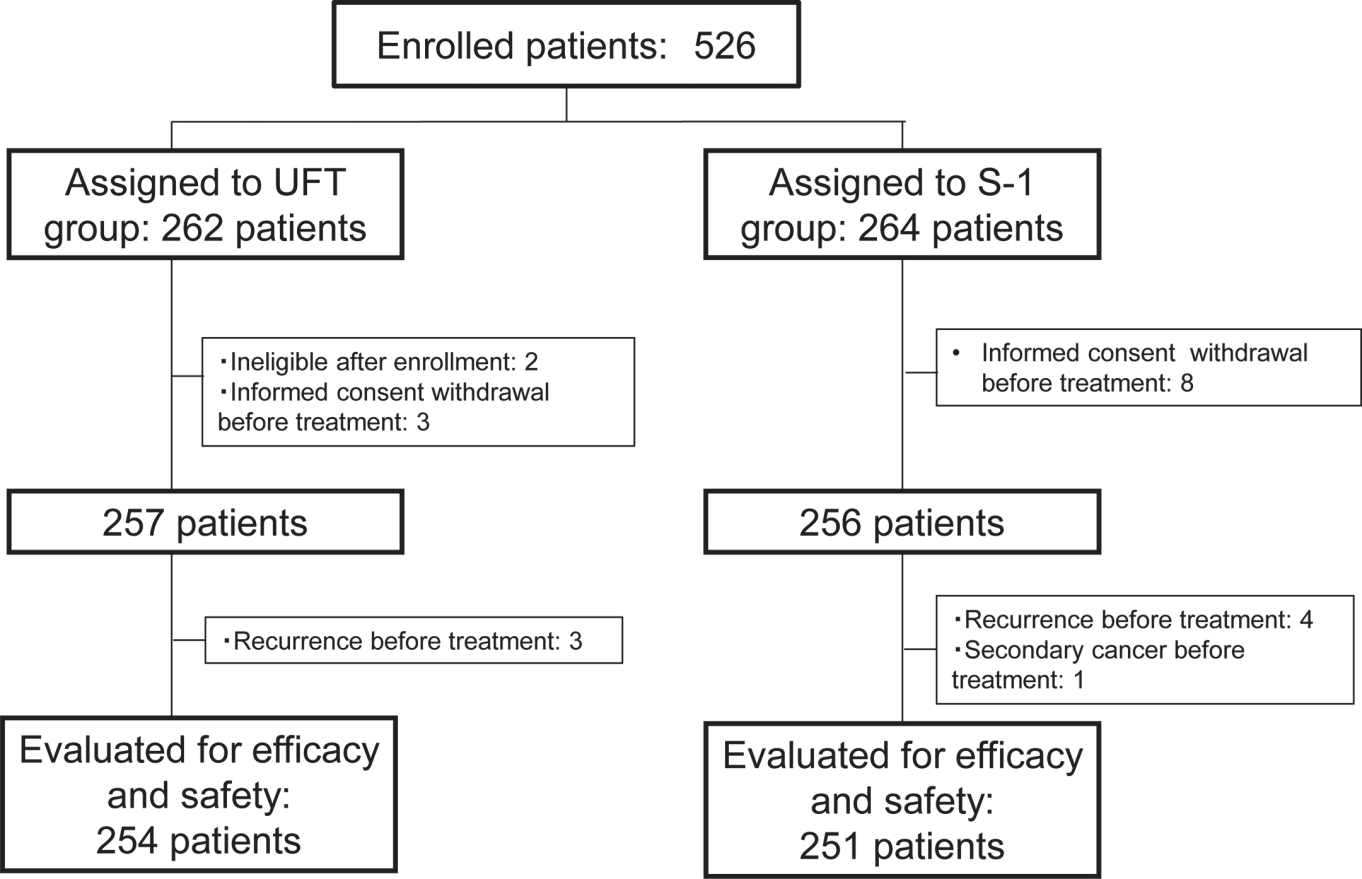
CONSORT diagram. ITT, Intention-to-treat.

From April 2006 through November 2008, a total of 526 patients were enrolled at 93 institutions: 262 patients were assigned to the UFT group and 264 to the S-1 group. Twenty-one patients were excluded because of ineligibility, retraction of informed consent, recurrence, or diagnosis of a secondary cancer before staring treatment. The other 505 patients were included in analysis (ITT) (Fig. 2).

Primary tumors were located in the hypopharynx in 71 patients (28.0%) in the UFT group and 71 (28.3%) in the S-1 group. The most common disease stage was IVA, diagnosed in 177 patients (69.7%) in the UFT group and 176 (70.1%) in the S-1 group. The initial curative treatment was surgery in 149 patients (58.7%) in the UFT group and 151 (60.2%) in the S-1 group. Patient characteristics did not differ significantly between the groups (Table 1).

**Table 1:** Patient characteristics.

		**UFT group (n = 254)**	**S-1 group (n = 251)**	**P value**
Age*		61 (29–75)	62 (26–75)	0.652†
Gender	Male	215 (84.6%)	209 (83.3%)	0.673
	Female	39 (15.4%)	42 (16.7%)	
Primary site	Maxillary sinus	18 (7.1%)	21 (8.4%)	0.988
	Oral cavity	61 (24.0%)	59 (23.5%)	
	Oropharynx	55 (21.7%)	53 (21.1%)	
	Hypopharynx	71 (28.0%)	71 (28.3%)	
	Larynx	49 (19.3%)	47 (18.7%)	
Stage	III	66 (26.0%)	67 (26.7%)	0.792
	IV A	177 (69.7%)	176 (70.1%)	
	IV B	11 (4.3%)	8 (3.2%)	
PS	0	234 (92.1%)	231 (92.0%)	0.969
	1	20 (7.9%)	20 (8.0%)	
Curative treatment	Surgery	149 (58.7%)	151 (60.2%)	0.732
	RT/CRT after surgery	86/149 (57.7%)	92/151(60.9%)	
	RT/CRT	105 (41.3%)	100 (39.8%)	
	Surgery after RT/CRT	34/105 (32.4%)	40/100 (40.0%)	

*Median (range),

†Age, Wilcoxon rank sum test; other, Chi-squared test

Abbreviations: RT, radiotherapy. CRT, chemoradiotherapy, PS: Eastern cooperative Oncology Group (ECOG) Performance Status

### Study Treatments

The median relative duration of treatment (ratio of actual duration of treatment to planned duration of treatment) was 1.0 in the UFT group and 0.9 in the S-1 group. The numbers of patients who were receiving the study treatment after 3 months, 6 months, and 12 months were respectively 205 (80.7%), 173 (68.1%), and 148 (58.3%) in the UFT group and 183 (72.9%), 149 (59.4%), and 109 (43.4%) in the S-1 group (Table 2). In the UFT group, the reasons for discontinuing treatment were the development of recurrence or secondary cancer in 49 patients, the physician’s judgment (mainly because of adverse events) in 33, a rest period exceeding 28 days in 13, death in 1, and other reasons in 17. In the S-1 group, the reasons for discontinuing treatment were the development of recurrence or secondary cancer in 33 patients, the physician’s judgment (mainly because of adverse events) in 52, a rest period exceeding 28 days in 40, death in 1, and other reasons in 35. Some of the reasons overlapped in both groups.

**Table 2:** Treatment completion rates.

	**UFT (n = 254)**		**S-1 (n = 251)**	
	**n**	**(%)**	**n**	**(%)**
3 months	205	(80.7)	183	(72.9)
6 months	173	(68.1)	149	(59.4)
12 months	148	(58.3)	109	(43.4)

### Survival

The median follow-up time was 1356 days (range, 35 to 2116).

**Fig 3:**
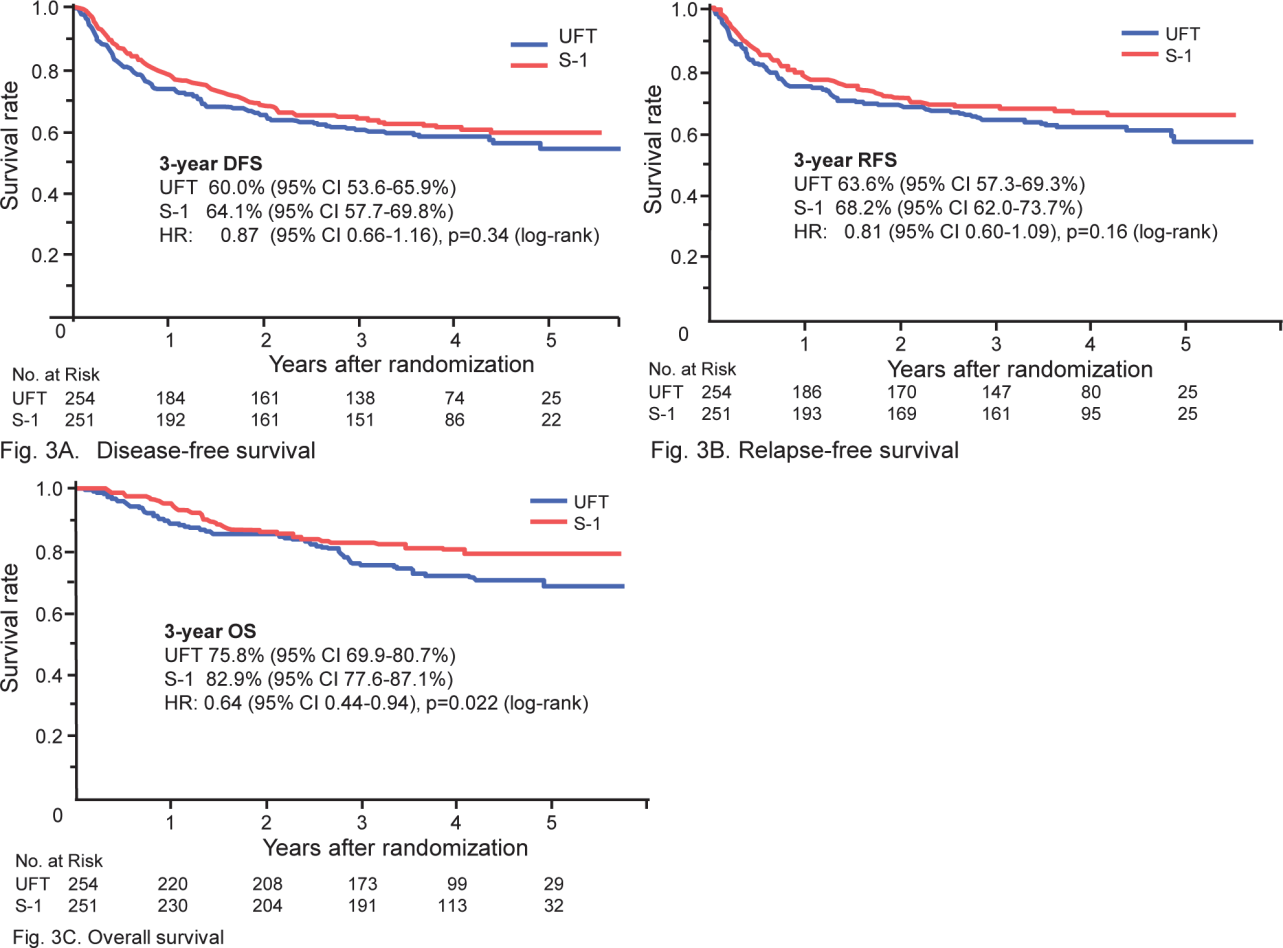
Kaplan-Meier estimates of disease-free survival (A), relapse-free survival (B), and overall survival (C).

**DFS.** The number of events was 107 in the UFT group and 97 in the S-1 group. The 3-year DFS rate was 60.0% (95% CI, 53.6% to 65.9%) in the UFT group and 64.1% (95% CI, 57.7% to 69.8%) in the S-1 group. The HR was 0.87 (95% CI, 0.66 to 1.16), and DFS did not differ significantly between the groups (p = 0.34) (Fig. 3A). Recurrence developed in 81 patients in the UFT group and 73 patients in the S-1 group. The initial type of recurrence was respectively local recurrence, lymph-node metastasis, and distant metastasis in 36, 22, and 35 patients in the UFT group and in 42, 20, and 25 patients in the S-1 group. Secondary cancers developed in 23 patients in the UFT group and 24 patients in the S-1 group.

**RFS.** The 3-year RFS rate was 63.6% (95% CI, 57.3% to 69.3%) in the UFT group and 68.2% (95% CI, 62.0% to 73.7%) in the S-1 group. The HR was 0.81 (95% CI, 0.60 to 1.09). RFS did not differ significantly between the groups (p = 0.16) (Fig. 3B).

**OS.** The 3-year OS rate was 75.8% (95% CI, 69.9% to 80.7%) in the UFT group and 82.9% (95% CI, 77.6 to 87.1%) in the S-1 group. The HR was 0.64 (95% CI, 0.44 to 0.94). OS was significantly better in the S-1 group than in the UFT group (p = 0.022) (Fig. 3C).

**Fig 4:**
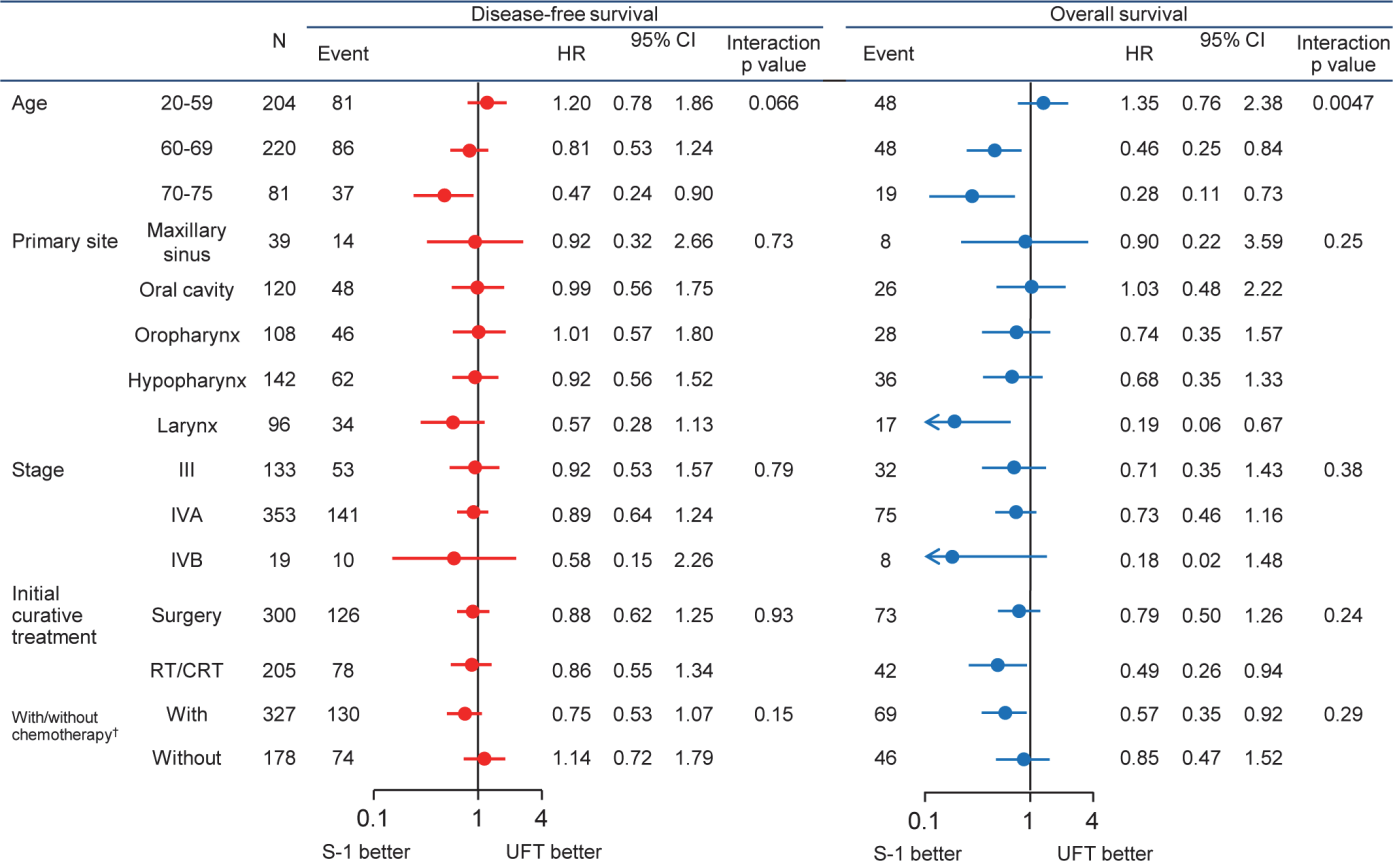
Subgroup Analysis: disease-free survival and overall survival. With/without chemotherapy†: Whether chemotherapy was received at curative treatment.

**Fig 5:**
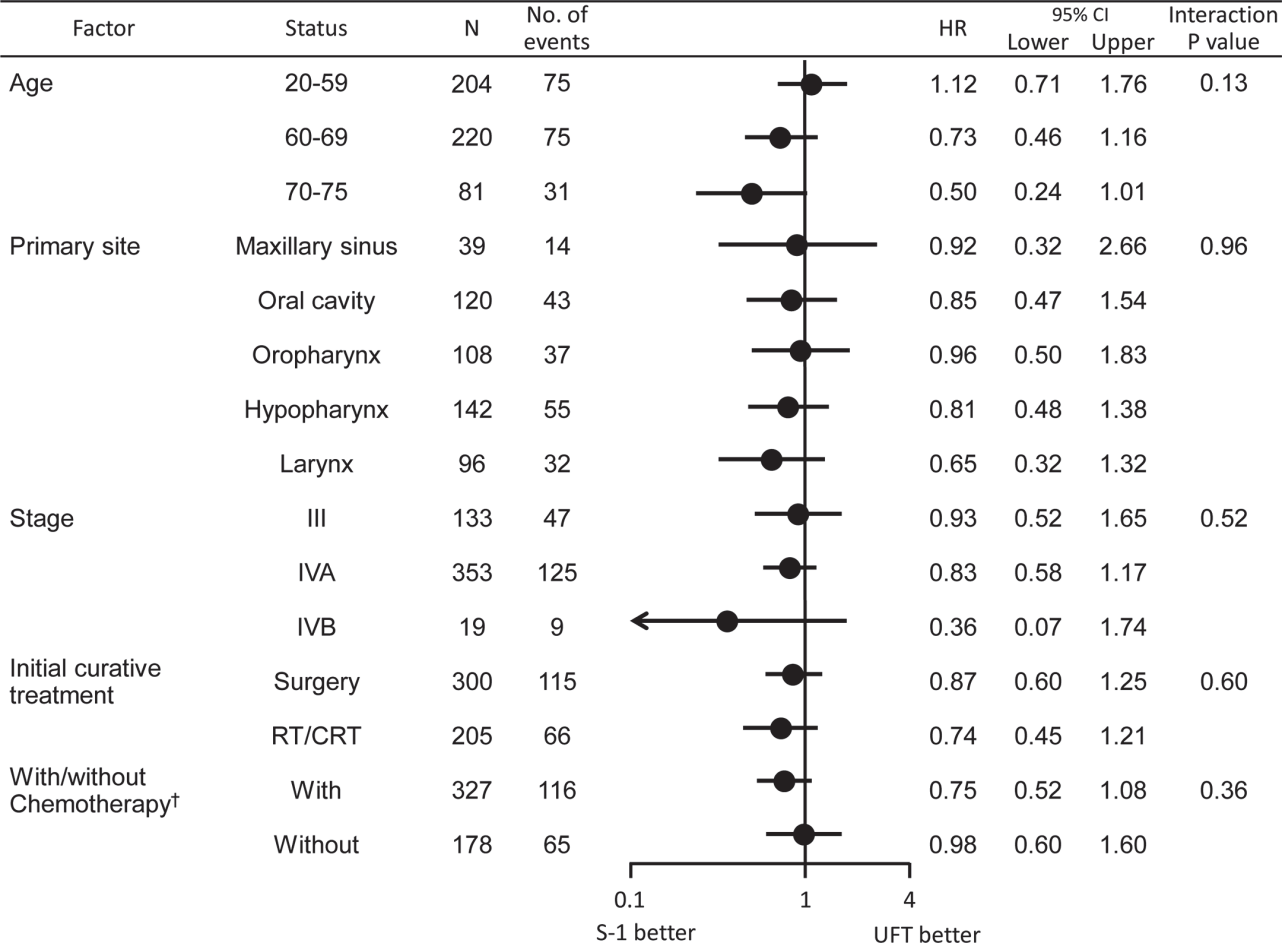
Subgroup analysis: relapse-free survival. With/without chemotherapy†: Whether chemotherapy was received at curative treatment

**Subgroup analysis.** Apart from an interaction between age and treatment, there were no other significant interactions. Although the interaction between primary tumor site and treatment was not significant, primary tumors arising in the larynx were associated with a trend toward better DFS and OS in the S-1 group (Fig. 4). RFS showed a similar trend (Fig. 5).

### Adverse Events

The following grade 3 or higher adverse events (hyperpigmentation, grade 2) occurred at an incidence of 1% or higher: elevated aspartate aminotransferase levels in 3 patients (1.2%), fatigue in 3 (1.2%), and anorexia in 3 (1.2%) in the UFT group and leukopenia in 13 patients (5.2%), neutropenia in 9 (3.6%), thrombocytopenia in 5 (2.0%), anemia in 5 (2.0%), elevated aspartate aminotransferase levels in 4 (1.6%), fatigue in 5 (2.0%), anorexia in 4 (1.6%), rash/desquamation in 5 (2.0%), hyperpigmentation in 9 (3.6%), and mucositis/stomatitis in 6 (2.4%) in the S-1 group. The incidences of the following adverse events differed significantly between the groups: leukopenia, neutropenia, thrombocytopenia, hyperpigmentation, and mucositis/stomatitis (Table 3). There were no treatment-related deaths in either group.

**Table 3:** Adverse events.

	**UFT (n = 254) **				**S-1 (n = 251) **				**p-value^†^**
	**All grade**		**Grade 3+4**		**All grade**		**Grade 3+4**		
	**n**	**(%)**	**n**	**(%)**	**n**	**(%)**	**n**	**(%)**	
Leukopenia	40	(15.7)	2	(0.8)	91	(36.3)	13	(5.2)	0.004
Neutropenia	14	(5.5)	0	(0.0)	58	(23.1)	9	(3.6)	0.002
Thrombocytopenia	17	(6.7)	0	(0.0)	32	(12.7)	5	(2.0)	0.030
Anemia	33	(13.0)	1	(0.4)	70	(27.9)	5	(2.0)	0.121
Total bilirubin increase	17	(6.7)	1	(0.4)	45	(17.9)	2	(0.8)	0.622
AST increase	23	(9.1)	3	(1.2)	25	(10.0)	4	(1.6)	0.723
ALT increase	14	(5.5)	2	(0.8)	20	(8.0)	2	(0.8)	1.000
Fatigue	34	(13.4)	3	(1.2)	79	(31.5)	5	(2.0)	0.502
Anorexia	34	(13.4)	3	(1.2)	74	(29.5)	4	(1.6)	0.723
Weight loss	14	(5.5)	1	(0.4)	28	(11.2)	1	(0.4)	1.000
Rash/desquamation	6	(2.4)	1	(0.4)	40	(15.9)	5	(2.0)	0.121
Hyperpigmentation	3	(1.2)	0	(0.0)	48	(19.1)	9*	(3.6) *	0.002*
Diarrhea	7	(2.8)	1	(0.4)	24	(9.6)	2	(0.8)	0.622
Mucositis/Stomatitis	5	(2.0)	0	(0.0)	32	(12.7)	6	(2.4)	0.015
Nausea	8	(3.1)	0	(0.0)	27	(10.8)	0	(0.0)	-
Vomiting	1	(0.4)	0	(0.0)	10	(4.0)	0	(0.0)	-

† Fisher’s exact test

* Number of grade 2 events are indicated, and differences in the incidences of grade 2 are tested.

### Exploratory analysis

**Fig 6:**
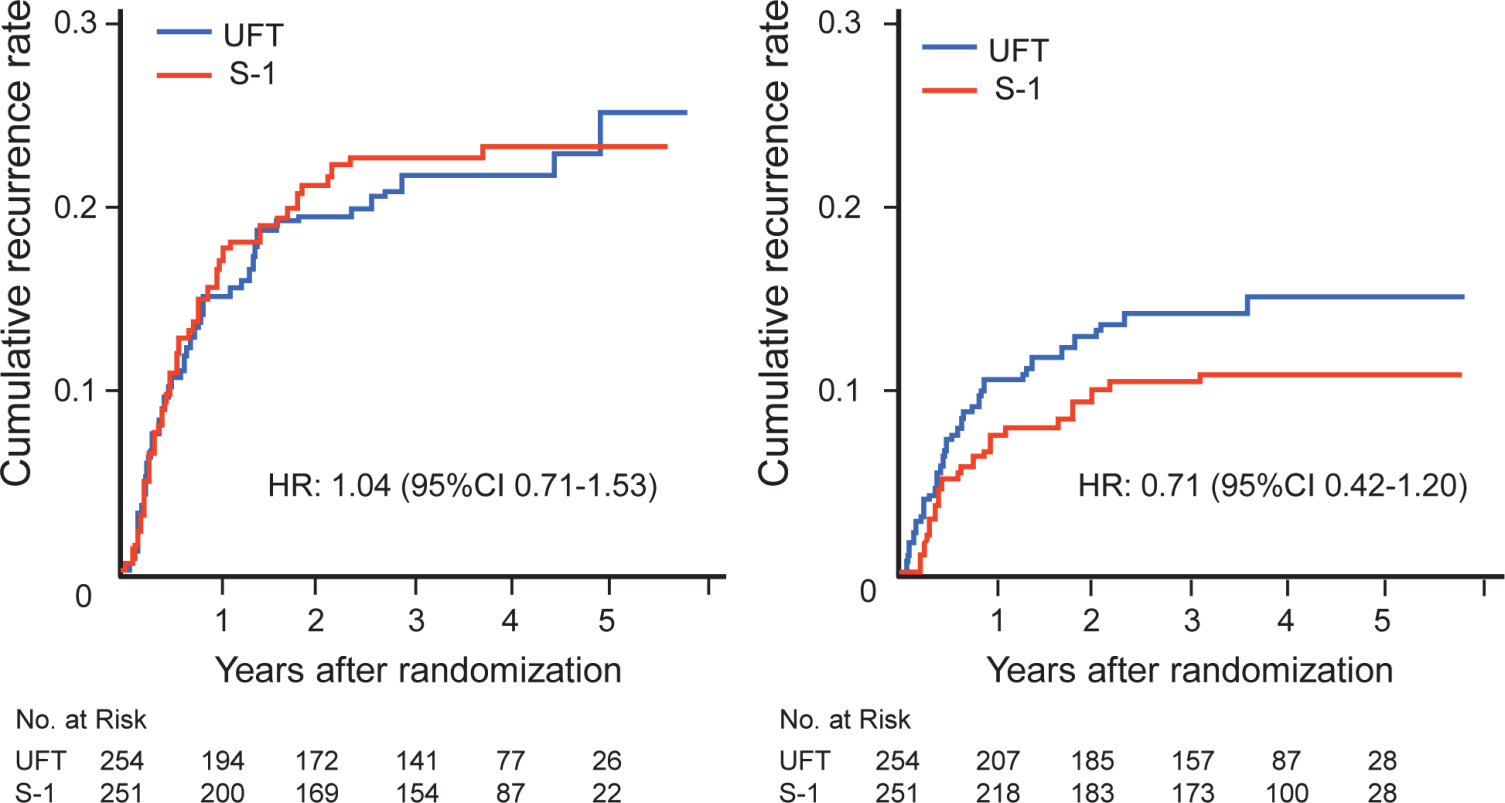
Cumulative rates of locoregional recurrence (A) and distant metastasis (B). (A) One patient with secondary cancer before locoregional recurrence was censored. (B) One patient with secondary cancer before distant metastasis was censored.

**Fig 7:**
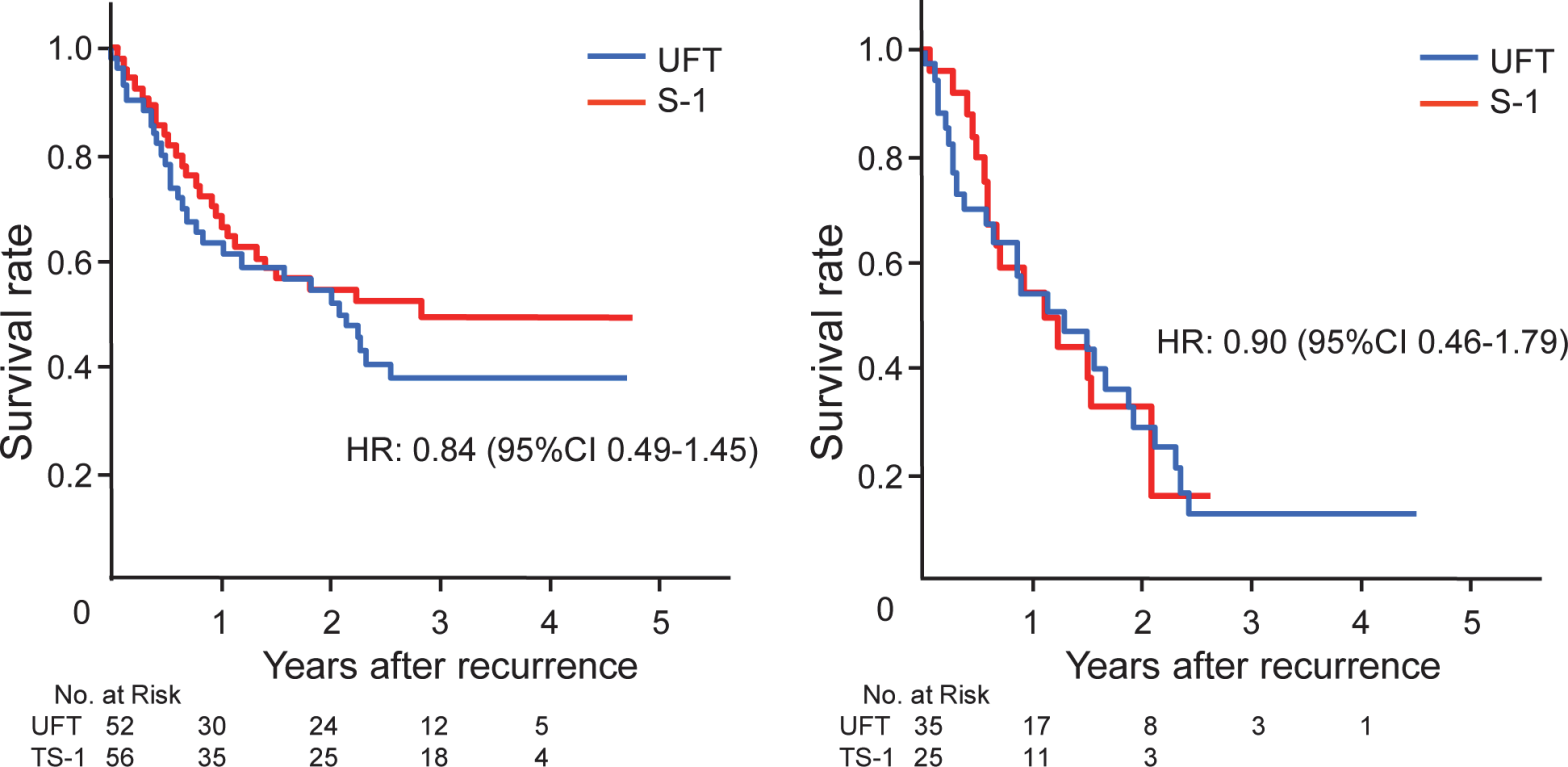
Survival from recurrence to death in patients with local recurrence/cervical lymph node recurrence (A) and in patients with distant metastasis (B).

**Locoregional recurrence rate and the time from locoregional recurrence to death.** Locoregional recurrence developed in 52 patients in the UFT group and 55 patients in the S-1 group. The HR was 1.04 (95% CI, 0.71 to 1.53) (Fig. 6A). When the survival time from recurrence was compared between the treatment groups, the HR for death was 0.84 (95% CI, 0.49 to 1.45) in the S-1 group as compared with the UFT group among patients who had local recurrence or lymph-node metastasis (Fig. 7A).

**Distant metastasis rate and the time from distant metastasis to death.** Distant metastasis was found in 34 patients in the UFT group and 25 patients in the S-1 group. The HR was 0.71 (95% CI, 0.42 to 1.20) (Fig. 6B). On analysis of the survival time from the development of distant metastasis to death, the HR for death was 0.90 (95% CI, 0.46 to 1.79) in the S-1 group as compared with the UFT group (Fig. 7B).

## Discussion

This study was designed to evaluate the effectiveness of adjuvant chemotherapy after curative treatment in patients with SCCHN. A meta-analysis by Pignon et al. did not provide convincing evidence of any benefits of adjuvant chemotherapy in patients with head and neck cancer [7]. Induction chemotherapy followed by radical therapy is expected for improvement of OS. However, no phase III trials have showed OS advantage of induction chemotherapy [24,25]. To our knowledge, the present study is the first phase III trial to demonstrate that adjuvant chemotherapy improved OS in patients who received curative treatment for stage III, IVA, or IVB SCCHN, as compared with a control group.

At time of planning this study, there was no standard treatment for SCCHN after curative therapy. Placebo was initially considered the best control treatment for a phase III trial of S-1. As mentioned in the Introduction section, however, the rate of distant metastasis was significantly lower in the UFT group (7.9%) than in the control group (14.6%, P = 0.034) in a previous multicenter study performed in Japan [18]. UFT was therefore been used for adjuvant therapy in many hospitals in Japan. Moreover, continuation of UFT treatment was requested by investigators from many hospitals when we designed the protocol and we realized that UFT was community-based standard therapy in Japan. UFT was therefore designated as control treatment for Japanese ethical reasons.

Although the DFS curve was consistently higher in the S-1 group than in the UFT group in our study, the primary end point of significantly better DFS in the S-1 group was not met. On the other hand, S-1 significantly improved OS as compared with UFT. One reason for the difference in OS may be that the incidence of distant recurrence was lower in the S-1 group (HR for distant metastasis, 0.71 [95% CI, 0.42 to 1.20]) than in the UFT group, most likely contributing to better survival in the S-1 group (Fig. 6B). Local and cervical lymph-node recurrence can sometimes be secondarily treated curatively by salvage therapy. However, secondary curative treatment of lesions in patients with distant metastasis remains difficult. Patients with distant metastasis have been reported to have poorer outcomes than those with local recurrence [26]. Therefore, prevention of distant recurrence is very important. After the study treatment was discontinued, 85 patients (33.5%) in the UFT group and 88 patients (35.1%) in the S-1 group received subsequent therapy, with no difference between the groups. Although it is difficult to explain why OS, but not DFS or RFS, was significantly better in S-1 group, adjuvant chemotherapy with S-1 appears to beneficially affect survival. Because assessments were presumed to be conducted during the same intervals in each arm, we believe that the impact of assessment interval on the results for DFS was minimal. However, a potential negative impact on DFS of using a predefined assessment interval of every 3 to 6 months, rather than every 6 months, cannot be ruled out.

The treatment completion rate in our study was 58.3% in the UFT group and 43.4% in the S-1 group. The rates of the following grade 3 or higher adverse events were significantly higher in the S-1 group: leukopenia (5.2%), neutropenia (3.6%), thrombocytopenia (2.0%), and mucositis/stomatitis (2.4%). Although the incidences of these adverse events were not high, S-1 was withdrawn at the discretion of the attending physician in 52 patients, suggesting that grade 2 or lower nonhematologic toxicity might have influenced the continuation of treatment. In a phase III study (ACTS-GC) in patients with gastric cancer, the rate of completing 12 months of treatment with S-1 was 65.8%, which was higher than the rate in our study. In addition, S-1 improved OS and RFS, as compared with surgery alone [15,16]. Our results suggest that the low-grade adverse events might have negatively affected compliance in patients with SCCHN, possibly leading to the relatively low treatment completion rate in this study.

Among factors potentially affecting survival in head and neck cancer, the presence of human papilloma virus (HPV) infection is known to be a determinant of outcomes in patients with oropharyngeal cancer[27]. Because this fact was not known at the time of planning our study in 2006, we unfortunately did not confirm the status of HPV infection in our subjects. However, because the primary tumor site was used as a stratification factor in our study, the proportions of patients with oropharyngeal cancer were similar in the treatment groups.

The main finding of our study was that single-agent chemotherapy with S-1, administered on an outpatient basis, improved OS after curative therapy as compared with the control group. Our results suggest that adjuvant chemotherapy with S-1 is one of the treatment options after curative therapy for locally advanced SCCHN. We anticipate that further evidence from controlled clinical studies will establish adjuvant chemotherapy after curative treatment as a new treatment strategy for patients with SCCHN.

## Supporting Information

S1 CONSORT Checklist (DOC)

S1 Protocol (DOCX)

S1 List List of IRB or IEC. (PDF)

S1 Table Criteria for temporary treatment withdrawal. (DOCX)

S2 Table Criteria for treatment resumption. (DOCX)

S3 Table Criteria for dose reduction. (DOCX)

S4 Table Levels for UFT dose reduction. (DOCX)

S5 Table Levels for S-1 dose reduction. (DOCX)
