# Supplementary material for: Randomized Phase III Trial of Adjuvant Chemotherapy with S-1 after Curative Treatment in Patients with Squamous-Cell Carcinoma of the Head and Neck (ACTS-HNC)
Source: PLoS One. 2015 Feb 11;10(2):e0116965. doi: 10.1371/journal.pone.0116965 (PMC4324826; doi:10.1371/journal.pone.0116965)
Supplement: S1 Protocol — (DOCX) [file pone.0116965.s002.docx]

Adjuvant Chemotherapy with TS-1 after Curative Therapy in Patients with Head and Neck Squamous Cell Cancer

― Multicenter Randomized Study ―

Protocol

Principal investigator: Masato Fujii

Division of Hearing and Balance Research, Clinical Research Center,

Tokyo Medical Center

Assistant principal investigator: Akira Kubota

Department of Head and Neck Surgery

Kanagawa Cancer Center

Version 1.0: January 6, 2006

Version 1.1: May 25, 2006

Version 1.2: December 1, 2006

Version 2.0: September 10, 2007

Version 2.1: February 1, 2011

Version 3.0: January 10, 2012

Confidentiality Statement:

This protocol contains confidential information that is the property of the Head and Neck Cancer Chemotherapy Study Group (HNCCSG) and must not be disclosed to anyone other than those directly involved in this study or ethics committees. This information may not be used for purposes other than performance or evaluation of the study without the advance written permission of the lead investigator.

All parties involved in this study will act in accordance with the Declaration of Helsinki and the Ethical Guidelines for Clinical Studies.

Table of Contents

[0. Schema 1](#_Toc363739518)

[1. Objective 2](#_Toc363739519)

[2. Background and Rationale 2](#_Toc363739520)

[3. Drug Information 5](#_Toc363739521)

[4. Diagnostic Criteria, Staging, and Classification 6](#_Toc363739522)

[5. Eligibility Criteria 7](#_Toc363739523)

[6. Enrollment and Randomization 10](#_Toc363739524)

[7. Treatment Plan 11](#_Toc363739525)

[8. Adverse Event Evaluation and Reporting 16](#_Toc363739526)

[9. Observations, Tests, Reported Parameters, and Schedule 17](#_Toc363739527)

[10. Target sample size and study period 23](#_Toc363739528)

[11. Definitions of Endpoints 23](#_Toc363739529)

[12. Statistical Considerations 24](#_Toc363739530)

[13. Independent Data Monitoring Committee 25](#_Toc363739531)

[14. Ethics-related items 26](#_Toc363739532)

[15. Financial Burden for the Study 28](#_Toc363739533)

[16. Protocol Amendments 29](#_Toc363739534)

[17. Completion and Early Termination of the Study 29](#_Toc363739535)

[18. References 31](#_Toc363739536)

# Schema

Follow-up period: 3 years after enrollment of the last subject

Outcome reporting: from discontinuation / completion of the protocol treatment to 3 years after enrollment of the last subject

Perform curative therapy

Squamous cell cancer of the head and neck

(Maxillary sinus, oral cavity, oropharynx, hypopharynx, larynx)

Stage III or Stage IV A or B

Capable of undergoing curative therapy

Test arm: 250 subjects

TS-1 80, 100, 120 mg/day

2 weeks on, 1 week off for 1 year

Control arm: 250 subjects

UFT 300, 400 mg/day

Daily administration for 1 year

Enrollment

(Enrollment period: April 2006 to March 2009)

Informed consent

Confirm tumor-free

(Within 3 months of completion of curative therapy)

Randomization

Adjustment factors (site, stage, type of curative therapy, study site)

Summary of study design

∙Phase: III ∙Randomization: yes (minimization)

∙Design features: 2-arm parallel design ∙Level of blinding: open-label

∙Type of control: active control (UFT)

# Objective

To perform a randomized comparison of the efficacy of TS-1 adjuvant chemotherapy and a UFT control in patients with clinical and/or pathological Stage III or Stage IV A or B squamous cell cancers of the head and neck at sites other than the oropharynx who have undergone standard curative therapy consisting of surgical therapy and radiotherapy (including combination chemotherapy and radiotherapy) and are confirmed tumor-free. The primary endpoint is disease-free survival, and the secondary endpoints are relapse-free survival, overall survival, and adverse events.

# Background and Rationale

2.1. Background

Advanced cancers of the head and neck have been treated with a combination of surgery and radiotherapy. Since the head and neck region plays an important role in daily activities and is also important cosmetically, combination radiotherapy and chemotherapy have recently been used for curative therapy to preserve organs and function.

However, curative therapy has not produced satisfactory results in cases of advanced cancer. Combining adjuvant chemotherapy with these treatments has also been studied, but has not yielded adequate results.

2.2. Adjuvant chemotherapy in the treatment head and neck cancer

In an Intergroup study (0034), subjects with Stage III or IV (M0) squamous cell cancer of the oral cavity, oropharynx, hypopharynx, and larynx that had undergone resection were randomized postoperatively to radiotherapy alone (224 subjects) or to chemotherapy followed by radiotherapy (222 subjects). A slight, but not significant difference was seen in 4-year survival rate and 4-year relapse-free survival rate, but the group receiving radiotherapy followed by chemotherapy showed a significantly lower (p=0.02) relapse rate of distant metastases (15%, 33/222) compared to the group receiving radiotherapy alone (23%, 51/224), suggesting that chemotherapy can potentially control distance metastases^1^. Bernier and colleagues^2^ compared two groups of patients with Stage III or IV locally advanced head and neck cancer that had undergone surgery and then received concurrent cisplatin and radiotherapy or radiotherapy alone; the researchers reported significantly superior results with concurrent treatment with radiotherapy and chemotherapy for both overall survival (hazard ratio=0.70; 95%CI, 0.52-0.95; p=0.04) and relapse-free interval (hazard ratio=0.75 ;95%CI, 0.56-0.99; p=0.02). Cooper and coworkers^3^ performed a comparison of chemoradiotherapy and radiotherapy alone as postoperative therapy in a high-risk group that had undergone total resection of head and neck cancers; although there was no difference in overall survival, Cooper et al obtained significantly superior results for disease-free survival (hazard ratio=0.78;95%CI, 0.61-0.99; p=0.04) and local control rate (hazard ratio=0.61;95%CI, 0.41-0.91; p=0.01) in the combined chemotherapy and radiotherapy group. Thus, these reports suggest that adding chemotherapy to postoperative radiotherapy improves prognosis.

Two large-scale studies have been reported in Japan. In one, squamous cell head and neck cancer patients with controlled primary lesions and neck metastases were either administered continuous treatment with bestatin 30 mg/day for 2 years or were assigned to a non-treatment group. Two-year survival rate and 2-year relapse-free rate were slightly higher with bestatin, but the difference was not significant. However, a subsequent stratification by disease site revealed a significantly higher survival rate for oral cancer in the bestatin-treated arm, suggesting that adjuvant chemotherapy with bestatin is useful^4^.

The other study, by the Head and Neck UFT Study Group, compared 300-400 mg/day for 1 year with a non-treatment group in patients who had undergone curative therapy for head and neck cancer. The subjects were 398 patients with Stage II, III, or IV squamous cell cancers of the head and neck without distant metastases who had undergone curative resection. The 3-year survival rate and 3-year relapse-free rate were both higher in the UFT group (77.9% and 73.4% in the UFT-treated group and 72.9% and 66.2% in the non-treatment group, respectively), although the difference was not significant. However, the distant relapse rate was significantly (p=0.034) lower in the UFT group (7.9% in the UFT group and 14.6% in the non-treatment group), suggesting that UFT is useful as adjuvant chemotherapy^5^.

These findings suggest the potential for adjuvant chemotherapy to be useful after curative therapy given the right disease and drugs administered.

2.3. TS-1

TS-1 (capsules containing a combination of tegafur, gimeracil and oteracil potassium) is an oral 5-FU antineoplastic developed in Japan. Tegafur (FT) is a prodrug of 5-FU. Gimeracil is a reversible competitive inhibitor of the rate-controlling catabolic enzyme of 5-FU, and oteracil potassium, which controls gastrointestinal toxicity, is a reversible inhibitor of a phosphorylating enzyme of 5-FU. Gimeracil and oteracil potassium are combined with tegafur to increase 5-FU blood concentration, thereby enhancing the antineoplastic effect, and to mitigate the accompanying increase in gastrointestinal toxicity. TS-1 was approved for gastric cancer in January 1999 and for head and neck cancer in April 2001. Compiling clinical results from early and late phase II studies administering TS-1 divided into 2 oral doses daily (equivalent to FT 80-150 mg/day) revealed a response rate of 34.1% (29/85 subjects) in 85 subjects evaluable for therapeutic effect^6,7^. These results are virtually identical to those obtained with conventional therapies for head and neck cancers and suggest that TS-1 would be extremely useful as adjuvant chemotherapy which requires long-term administration because TS-1 is an oral formulation that can be administered on an outpatient basis. TS-1 was subsequently approved for colorectal cancer, lung cancer, and inoperable or recurrent breast cancer.

2.4. Investigational plan

2.4.1. TS-1 dosing schedule

Because TS-1 shows a potent antineoplastic effect and elicits a large number of adverse drug reactions^8^, scheduling of feasible dosing periods has been a problem. When considering the feasibility of a TS-1 regimen, it is important to reduce the number of subjects discontinuing due to adverse drug reactions. A dosing schedule of TS-1 for 2 weeks and 1 week of rest was therefore seemed useful for avoiding adverse drug reactions or preventing them from becoming serious. In fact, in a study of advanced relapsed gastric cancer, albeit with a small number of subjects, Kimura and colleagues reported that 2 weeks of TS-1 with a 1-week rest period improved feasibility because it afforded the same antineoplastic effect as 4-week administration and 2-week rest in addition to a reduction in serious adverse drug reactions^9^. From 2002, we performed a study comparing the feasibility of 6 months of treatment with the standard TS-1 regimen (4-week administration and 2-week rest) and 2-week administration and 1-week rest in subjects who had undergone curative therapy for squamous cell head and neck cancer with the objective of studying the optimal dosing method for long-term administration of TS-1 as adjuvant chemotherapy for such cancers; the study confirmed that a regimen of 2-week administration 1-week rest was highly feasible in terms of the low incidence of adverse drug reactions and total dose administered^10^. While the suitable duration of treatment for this regimen is not known, studies performed to date administered UFT for 1 year (Head and Neck UFT Study) ^5^, 2 years for non-small cell lung cancer^11^, and 1 year for rectal cancer^12^. Based on this evidence, it was decided to administer a regimen of 2-week TS-1 administration and 1-week rest for 1 year to subjects who had undergone curative therapy for squamous cell head and neck cancer in the present study in order to study the usefulness of TS-1 as adjuvant chemotherapy.

2.4.2. Control arm

An observation-only group could be appropriate for the control arm in consideration of the fact that the usefulness of adjuvant chemotherapy has not yet been established in terms of overall survival or disease-free survival. However the use of some treatment was ethically warranted in this study conducted in patients with advanced cancers with poor prognosis. Candidate treatments for the control arm included the CF regimen (consisting of cisplatin and fluorouracil) and UFT (tegafur/uracil) therapy. UFT was selected because it is an oral preparation, like TS-1, and its use as postoperative adjuvant chemotherapy has been suggested to suppress distant metastases of head and neck cancers. In contrast, the CF regimen requires hospitalization and is administered over several cycles, which would likely lead to decreased compliance with the protocol-specified schedule. The UFT dosage and duration of treatment used in the previous study of UFT for head and neck cancers have been employed (i.e., 300 or 400 mg/day for 1 year). The use of UFT is justified, as its usefulness has recently been recognized again in postoperative adjuvant chemotherapy for various cancers. Specifically, use of UFT as postoperative adjuvant therapy has been reported to have resulted in significantly improved overall survival and relapse-free survival in patients with non-small cell lung cancer, rectal cancer, and gastric cancer, as compared with surgery-only^11,12,13)^, thereby adding to the evidence on its efficacy as adjuvant chemotherapy.

2.4.3. Significance of this study

In the treatment concept for advanced head and neck cancers, no optimal multidisciplinary treatment algorithm has been established based on study results available to date, even for curative local treatment. Thus, surgery, radiotherapy, or chemotherapy regimens may vary considerably among individual patients as a result of different decisions of attending physicians or different treatment protocols. However, increasing amounts of data have suggested that distant metastases are suppressed by systemic chemotherapy administered during curative therapy or postoperatively as adjuvant therapy. The present study is designed to compare the efficacy of TS-1 and UFT as adjuvant therapy after curative local treatment in patients with advanced head and neck cancers with clinically- or histopathologically-confirmed complete response (CR). The two fluorinated pyrimidines with partly different mechanisms of action will be compared in terms of reduction of distant metastases to investigate the state of the art in this study using a large-scale prospective cohort design.

# Drug Information

Information on the drugs used in the present study is summarized below. Refer to the most recent prescribing information for details.

3.1. TS-1 (tegafur, gimeracil, oteracil potassium combination drug; proprietary name, TS-1 Capsule; abbreviated TS-1)

3.1.1. Overview

TS-1 is an oral 5-FU antineoplastic developed in Japan. Tegafur (FT) is a prodrug of 5-FU. Gimeracil is a reversible competitive inhibitor of the rate-controlling catabolic enzyme of 5-FU, and oteracil potassium, which controls gastrointestinal toxicity, is a reversible inhibitor of a phosphorylating enzyme of 5-FU. Gimeracil and oteracil potassium are combined with tegafur to increase 5-FU blood concentration, thereby enhancing the antineoplastic effect, and to mitigate the accompanying increase in gastrointestinal toxicity. TS-1 has been approved for head and neck cancers, gastric cancer, colorectal cancer, non-small cell lung cancer, and inoperable or recurrent breast cancer, pancreatic cancer, and biliary tract cancer.

3.1.2. Pharmacokinetics

The AUC and Cmax of FT, gimeracil (CDHP), oteracil potassium (Oxo), and 5-FU increased dose-dependently after oral administration of TS-1. Plasma concentration rapidly achieved steady state with 32-40 mg/m^2^ of TS-1 twice daily. CDHP-induced DPD inhibition was reversible and was not intensified with daily dosing.

3.1.3. Toxicity

Refer to the most recent prescribing information.

3.1.4. Contraindications

A history of serious hypersensitivity to the ingredients of TS-1; serious myelosuppression; serious renal dysfunction; serious hepatic dysfunction; current treatment with another fluorinated pyrimidine antineoplastic; current treatment with flucytosine; pregnancy or potential pregnancy

3.1.5. Concomitant use contraindicated or requires caution

Concomitant use contraindicated: (1) fluorinated pyrimidine antineoplastics (combinations of fluorouracil, tegafur, and uracil; tegafur; doxifluridine; capecitabine; carmofur), (2) folinate, tegafur, uracil therapy; levofolinate, fluorouracil therapy, (3) fluorinated pyrimidine antifungals (flucytosine)

Concomitant use requires caution: phenytoin, warfarin potassium, other antineoplastics, radiotherapy, etc.

3.2. UFT (tegafur, uracil combination drug; proprietary name, UFT Capsule^®^; abbreviated UFT)

3.2.1. Overview

UFT is a 5-FU antineoplastic developed in Japan. FT is a prodrug of 5-FU, and the biogenic substance uracil is a reversible competitive inhibitor of the rate-controlling catabolic enzyme of 5-FU. Combining uracil with FT increases the blood 5-FU concentration, thereby enhancing the antineoplastic effect. UFT has been approved for head and neck cancer, gastric cancer, colorectal cancer, liver cancer, gallbladder and biliary tract cancer, pancreatic cancer, lung cancer, breast cancer, bladder cancer, prostate cancer, and uterine cancer.

3.2.2. Pharmacokinetics

When 3 UFT capsules were orally administered to cancer patients and the blood FT, 5-FU, and uracil concentrations were measured, FT concentration peaked 2 hours later and then gradually decreased. 5-FU and uracil concentrations both peaked 30 min after administration and then decreased.

3.2.3. Toxicity

Refer to the most recent prescribing information.

3.2.4. Contraindications

Serious myelosuppression; serious diarrhea; serious complicating infections; a history of serious hypersensitivity to the ingredients in UFT; current treatment with tegafur, gimeracil, oteracil potassium combination drug or less than 7 days since discontinuation; pregnancy or potential pregnancy

3.2.5. Concomitant use contraindicated or requires caution

Concomitant use contraindicated: (1) tegafur, gimeracil, oteracil potassium combination drug(TS-1)

Concomitant use requires caution: (1) phenytoin, (2) warfarin potassium, (3) other antineoplastics, radiotherapy, etc.

# Diagnostic Criteria, Staging, and Classification

4.1 Diagnostic criteria

General Rules for Clinical Studies on Head and Neck Cancer, October 2005 (The Fourth Edition) by the Japan Society for Head and Neck Cancer was used for diagnostic criteria, staging, classification, etc. The histologic grade was used.

4.2. Staging

UICC TNM Version 6 will be used.

The staging table is shown below.

| Stage | T | N | M |
| --- | --- | --- | --- |
| Stage 0 | Tis | N0 | M0 |
| Stage I | T1 | N0 | M0 |
| Stage II | T2 | N0 | M0 |
| Stage III | T3 | N0, N1 | M0 |
|  | T1, T2 | N1 | M0 |
| Stage IV A | T1, T2, T3 | N2 | M0 |
|  | T4a | N0, N1, N2 | M0 |
| Stage IV B | T4b | Any N | M0 |
|  | Any T | N3 | M0 |
| Stage IV C | Any T & N | | M1 |

# Eligibility Criteria

5.1. Inclusion criteria

Patients satisfying all of the following conditions at consent will be enrolled in the study.

1) Can be enrolled within 3 months after completion of first curative therapy

2) Histological diagnosis of squamous cell cancer

3) Primary site is the maxillary sinus, oral cavity, oropharynx, hypopharynx, or larynx (except T3N0 of the glottis)

4) Clinical or pathological Stage III or Stage IVA or IVB that has undergone first curative therapy (surgery or radiotherapy (including combination chemotherapy and radiotherapy)

Subjects will be excluded if they have lower Level IV (supraclavicular fossa) or Level V metastases pretreatment or in resected specimens.

5) Tumor eradication (primary tumor and lymph node metastases) is confirmed within 3 months of completion of first curative therapy (refer to “5.3. Definition of tumor eradication by curative therapy”).

6) Did not receive antineoplastic therapy between the completion of the first curative therapy and study enrollment.

7) 20 to 75 years of age at consent

8) Performance status (PS) of 0 or 1

9) Capable of taking oral medications.

10) Has the following levels of bone marrow, hepatic, and renal function within 14 days prior to study enrollment.

| WBC | ≥ 3,500 /mm^3^, < 12,000 /mm^3^ |
| --- | --- |
| Neut | ≥ 2,000 /mm^3^ |
| PLT | ≥ 100,000 /mm^3^ |
| Hb | ≥ 9.0 g/dL |
| T.Bil | < 1.5 mg/dL |
| AST∙ALT | < 100 IU/L |
| Cr | < 1.2 mg/dL |

11) Has not had multiple primary cancers within the last 3 years

12) Has personally given written informed consent.

5.2. Exclusion criteria

Patients to whom any of the following conditions apply at consent will be excluded from the study.

1) A history of hypersensitivity to the drugs used in this study or analogues of them

2) Use of flucytosine (concomitant use contraindicated)

3) Use of flucytosine (concomitant use contraindicated), phenytoin (concomitant use requires caution), or warfarin potassium (concomitant use requires caution) required during the protocol treatment period.

4) Complicating infections requiring treatment

5) Gastrointestinal diseases (e.g., intestinal paresis and intestinal obstruction) requiring treatment

6) Interstitial pneumonia or pulmonary fibrosis

7) Poorly controlled diabetes mellitus

8) Poorly controlled heart disease

9) Serious hepatic dysfunction

10) Diarrhoea (watery stools)

11) Potentially pregnant or intent to become pregnant, pregnant, or nursing (women)

12) Does not consent to using contraception during the study (men)

13) Deemed otherwise unsuitable for the study by the investigator

5.3. Definition of tumor eradication by curative therapy

A diagnosis of “tumor eradication” will be rendered in the following instances.

1) For surgery

There are no cancer cells in the margins of specimens excised from the primary site pathologically and there are no new findings on diagnostic imaging (simple chest radiography, ultrasonography, CT, MRI, PET, etc.)

In addition, subjects can be enrolled when there are extranodal infiltration and multiple metastasized lymph nodes in the specimen excised from the primary site, postoperative radiotherapy (including concomitant chemotherapy and radiotherapy) is applied

and tumor eradication is confirmed on diagnostic imaging 1 month after completion of therapy.

Specimen: negative margins

Confirmed tumor free (imaging)

Surgery

Eligible for enrollment during this period

(NAC)

3 months

Radiation to neck

Surgery

Confirmed tumor free (imaging)

Eligible for enrollment during this period

3 months

1 month

Extranodal infiltration and multiple

metastasized lymph nodes in the specimen

2) For radiotherapy (including concomitant chemotherapy and radiotherapy)

Eradication of the primary lesion and lymph node metastases are confirmed on diagnostic imaging (simple chest radiography, ultrasonography, CT, MRI, PET, etc.) starting 1 month or more after curative therapy.

When residual tumor is suspected at the primary lesion from inspection or diagnostic imaging 1 month or more after completion of curative therapy, the site will be confirmed to be tumor free pathologically (by biopsy).

Confirmed tumor free

Imaging (primary lesion and lymph node metastases)

Radiotherapy

(Including concomitant chemotherapy)

Biopsy (primary lesion: residual tumor suspected)

Eligible for enrollment during this period

1 month

3 months

3) Cervical lymph nodes

When cervical lymph nodes are less than 1 cm and residual tumor is suspected, the diagnostic method including fine needle aspiration cytology will be performed to confirm the presence of residual tumor.

A subject can be enrolled when residual metastasized cervical lymph nodes are suspected after radiotherapy (including concomitant chemotherapy and radiotherapy), if neck dissection is performed as first-line therapy, and the site is considered tumor free.

Radiotherapy

tumor free

確認

登録可能期間

3

ヶ月

放射線

（化学療法併用可）

頸部転移リンパ節あり

1cm

未満で、残存が疑われる場合は、

穿刺吸引細胞診などで残存の有無を確認する）

頸部

郭清

tumor free

確認

登録可能期間

3

ヶ月

1cm

（頸部リンパ節が

未満で、残存が疑われる場合は、

頸部転移リンパ節あり

穿刺吸引細胞診などで残存の有無を確認する）

放射線

（化学療法併用可）

頸部

郭清

Confirmed tumor free

Eligible for enrollment during this period

Neck dissection

3 months

Metastasized cervical lymph nodes present

(Including concomitant chemotherapy)

(When cervical lymph nodes are less than 1 cm and residual malignancy is suspected, the diagnostic method including fine needle aspiration cytology will be performed to confirm the presence of residual malignancy.)

4) Tumor markers

Tumor eradication will not be determined solely on the basis of tumor markers.

5.4. Definition of tumor eradication confirmation date

When multiple diagnostic methods are used, the “tumor eradication confirmation date” will be the date the results of the last test are interpreted.

5.5. Definition of curative therapy completion date

The “curative therapy completion date” is the date of completion of the therapy that led to the permanent cure which includes not only the surgery or radiotherapy that was the (main) curative therapy, but also the series of treatments performed before and after.

# Enrollment and Randomization

Centralized enrollment of study sites and subjects will be performed by the TRI Center (referred to as the Data Center hereinbelow). The Data Center will be located in the Translational Research Informatics Center of the Foundation for Biomedical Research and Innovation.

6.1. Randomization method and randomization adjustment factors

Subjects will be randomized to the test arm (TS-1-treated arm) or control arm (UFT-treated arm). Randomization will be performed by the minimization method, using the 4 factors of (1) primary disease site, (2) stage (III, IVA, IVB), (3) type of curative therapy (surgery or radiotherapy), and (4) study site.

# Treatment Plan

7.1. Protocol treatment

After completion of enrollment with the Data Center, the test treatment (TS-1) or the control treatment (UFT) will be started within 3 months after completion of curative therapy. The protocol treatment will in principle be performed on an outpatient basis.

7.1.1. Control treatment arm (UFT treated arm)

1) Treatment regimen

UFT will be administered daily by the oral route for 1 year. The daily dose (300 mg or 400 mg), based on body surface, will be divided into 2 doses daily (after breakfast and dinner) or 3 doses daily (after breakfast, lunch, and dinner). After completion of UFT treatment, subjects will be followed for up to 5 years (for 3 years after enrollment of the last subject), and antineoplastic therapy will not be performed until metastases or relapse are confirmed.

Daily oral UFT treatment for 1 year

Day 1 1 year

UFT dose and administration method

| Body surface area | Single dose at treatment initiation (FT equivalent) | Daily dose  (FT equivalent) | Breakfast | Lunch | Dinner |
| --- | --- | --- | --- | --- | --- |
| < 1.5 m^2^ | 100 mg | 300 mg | 100 mg | 100 mg | 100 mg |
|  | 150 mg (granules) |  | 150 mg | - | 150 mg |
| ≥ 1.5 m^2^ | 200 mg | 400 mg | 200 mg | - | 200 mg |

2) UFT specifications

UFT capsules: 100 mg FT equivalent per capsule

UFT E granules: 200 mg FT equivalent per gram

7.1.2. Experimental treatment arm (TS-1-treated arm)

1) Treatment regimen

TS-1 will be administered by the oral route daily for 14 days (Day 1-14) with a 7 day rest period (Day 15-21). The daily dose (80 mg, 100 mg, or 120 mg), which is based on body surface, will be divided into 2 doses daily (after breakfast and dinner) This 21-day (3-week) period will comprise 1 cycle and will be repeated for 1 year from the protocol treatment initiation day (about 18 cycles will be administered). New cycles will not be started after 1 year has elapsed from the protocol treatment initiation date. After completion of UFT treatment, subjects will be followed for up to 5 years (for 3 years after enrollment of the last subject), and antineoplastic therapy will not be performed until metastases or relapse are confirmed.

Daily oral TS-1 treatment for 14 days

7-day rest period

Day 1 Day 14 Day 21

TS-1 dose and administration method

| Body surface area | Single dose at treatment  initiation (FT equivalent) | Daily dose  (FT equivalent) | Breakfast | Lunch | Dinner |
| --- | --- | --- | --- | --- | --- |
| < 1.25 m^2^ | 40 mg/dose | 80 mg | 40 mg | - | 40 mg |
| ≥ 1.25 m^2^ to <1.5 m^2^ | 50 mg/dose | 100 mg | 50 mg | - | 50 mg |
| ≥ 1.5 m^2^ | 60 mg/dose | 120 mg | 60 mg | - | 60 mg |

2) TS-1 specifications

TS-1 Capsules 20: 20 mg FT equivalent per capsule

TS-1 Capsules 25: 25 mg FT equivalent per capsule

7.2. Criteria for changing dose and schedule (Rest days or reduced dose days will be recorded in the chart without fail.)

The following terminology will be used for change criteria.

Rest: A temporary holiday during which drug is not administered during the protocol treatment dosing period after which treatment can be resumed if the conditions are satisfied.

Dose reduction: Administration of less than the specified dose

Discontinuation: The protocol treatment is discontinued, and treatment will not be resumed during the subject’s participation in the study.

7.2.1. Rest criteria and resumption criteria

When starting a TS-1 cycle (after the rest period from the previous cycle) and when performing tests during UFT treatment, it will be confirmed that all test values satisfy the dosing criteria before resuming treatment. When the dosing criteria are not satisfied, treatment will be withdrawn and resumed according to the following criteria.

1) Rest criteria

If the following adverse events which apply to the rest criteria occur during the drug treatment period, drug treatment will be immediately withdrawn, the (sub)investigator will wait until the values return to at least the treatment resumption criteria before resuming treatment.

The (sub)investigator will decide whether or not to withdraw treatment when rest criteria apply to the laboratory values of subjects whose baseline values are close to the rest criteria.

Rest criteria *Non-hematologic toxicities: Adverse events from sections of the CTCAE other than Blood/Bone Marrow

| Parameter | | Criterion for prescribing rest |
| --- | --- | --- |
| Hematologic toxicities | WBC | < 3,000 /mm^3^ |
|  | Neut | < 1,500 /mm^3^ |
|  | PLT | < 75,000 /mm^3^ |
|  | Hb | < 9.0 g/dL |
| Non-hematologic toxicities  * | T.Bil | ≥ 2.0 mg/dL |
|  | AST⋅ALT | ≥ 100 IU/L |
|  | Cr | ≥ 1.2 mg/dL |
|  | Other adverse events | ≥ Grade 2 |
| Other instances in which the investigator determines that rest is necessary because of adverse events, etc., in order to continue the protocol treatment. | | |

2) Treatment resumption criteria

(1) UFT-treated arm

After withdrawing treatment, the investigator will wait until the treatment resumption parameters return to normal to resume treatment. The dose at treatment resumption will be determined in accordance with the dose reduction criteria (refer to 7.2.2.). When the rest period exceeds 28 days due to adverse events, the subject’s protocol treatment will be discontinued.

(2) TS-1-treated arm

After a rest of at least 1 week in the TS-1-treated arm, it will be confirmed that the following treatment resumption criteria have been satisfied before resuming TS-1 treatment. The dose at treatment resumption will be determined in accordance with the dose reduction criteria (refer to 7.2.2.). When the rest period exceeds 28 days due to adverse events, the subject’s protocol treatment will be discontinued.

Example) When rest starts on Day 8 of the first cycle

after at least 1 week of rest, it is confirmed if treatment can be resumed.

→*If treatment can be resumed: the second cycle is started.

*If treatment cannot be resumed: Treatment resumption is postponed until the resumption criteria are satisfied.

However, when the rest period exceeds 28 days, the protocol treatment will be discontinued and only a follow-up will be performed because the discontinuation apply (refer to “7.4 Follow-up at discontinuation of protocol treatment”).

Treatment resumption criteria

*Non-hematologic toxicities: Adverse events from sections of the CTCAE other than Blood/Bone Marrow

| Parameter | | Treatment resumption criterion |
| --- | --- | --- |
| Hematologic toxicities | WBC | ≥ 3,000 /mm^3^ |
|  | Neut | ≥ 1,500 /mm^3^ |
|  | PLT | ≥ 100,000 /mm^3^ |
|  | Hb≥ | ≥ 9.0 g/dL |
| Non-hematologic toxicities* | T.Bil | < 1.5 mg/dL |
|  | AST⋅ALT | < 100 IU/L |
|  | Cr | < 1.2 mg/dL |
|  | Other adverse events | ≤ Grade 1 |

7.2.2. Dose reduction criteria

When the following adverse event criteria apply during a rest period, treatment will be resumed according to the dose reduction criteria.

| Adverse event criteria for reducing dose | | |
| --- | --- | --- |
| Hematologic toxicities | WBC | < 2,000 /mm^3^ (≥ Grade 3) |
|  | Neut | < 1,000 /mm^3^ (≥ Grade 3) |
|  | PLT | < 50,000 /mm^3^ (≥ Grade 3) |
|  | Hb | < 8.0 g/dL (≥ Grade 3) |
| Non-hematologic toxicities* | T.Bil | ≥ 3 mg/dL |
|  | AST⋅ALT | ≥ 150 IU/L |
|  | Cr | ≥ 1.2 mg/dL |
|  | Other adverse events | ≥ Grade 3 |
| Other instances in which the investigator determines that dose reduction is necessary because of adverse events, etc., in order to continue the protocol treatment. | | |

* Non-hematologic toxicities: Adverse events from sections of the CTCAE other than Blood/Bone Marrow

Dose reduction will be performed according to the following dose reduction criteria.

1) TS-1 dose reduction criteria

Dose reduction will be performed 1 level at a time, and the minimum dose will be 80 mg/day.

| Body surface area | Starting dose  (FT equivalent) | TS-1 dose level | | |
| --- | --- | --- | --- | --- |
|  |  | 1st dose reduction | 2nd dose reduction | 3rd dose reduction |
| < 1.25 m^2^ | 80 mg/day | Discontinuation | - | - |
| ≥ 1.25 m^2^ to < 1.5 m^2^ | 100 mg/day | 80 mg/day | Discontinuation | - |
| ≥ 1.5 m^2^ | 120 mg/day | 100 mg/day | 80 mg/day | Discontinuation |

2) UFT dose reduction criteria

The minimum dose will be 300 mg/day.

| Body surface area | Starting dose  (FT equivalent) | UFT dose level | |
| --- | --- | --- | --- |
|  |  | 1st dose reduction | 2nd dose reduction |
| < 1.5 m^2^ | 300 mg/day | Discontinuation | - |
| ≥ 1.5 m^2^ | 400 mg/day | 300 mg/day | Discontinuation |

7.2.3. Criteria for dose elevation after administration of reduced dose

Dose will not be increased for subjects receiving a reduced dose, even if the relevant adverse events return to normal.

7.3. Discontinuation of protocol treatment

The protocol treatment will be discontinued if any of the following conditions apply.

1) Confirmed metastasis or relapse of primary disease

2) Confirmed secondary cancer (double cancer)

3) Subject has died.

4) The TS-1 dose is < 80 mg/day or the UFT dose is < 300 mg/day, as a result of applying the dose reduction criteria

5) The rest period exceeds 28 days.

6) The subject withdraws consent for the protocol treatment (TS-1 or UFT treatment).

7) The subject’s ability to keep appointments is compromised by transferring hospitals, etc.

8) The subject is found to be ineligible after enrollment.

9) The (sub)investigator otherwise determines that continuation of the protocol treatment is compromised.

7.4. Follow-up at discontinuation of protocol treatment

Except for the subjects listed below, every effort will be made to follow-up for survival, metastasis, and relapse all of the subjects who discontinued the protocol treatment, based on the provisions in 7.3. If metastasis or relapse is confirmed, only outcome will be followed up.

1) The subject died.

2) The subject withdrew consent to be followed up.

3) The subject was found to be ineligible after beginning the protocol treatment.

4) Follow-up was compromised for some other reason.

Follow-up after treatment discontinuation will be performed every 3-6 months, counting from the starting day of the protocol treatment.

7.5. Concomitant and supportive therapy

7.5.1. Concomitant therapy that will not be permitted (prohibited concomitant therapy)

None of the following therapies will be performed during the protocol treatment or follow-up until relapse or secondary cancer is confirmed.

1) Antineoplastics other than the protocol treatment (including immunotherapy) and use of TS-1 or UFT during follow-up

2) Use of the antifungal flucytosine, phenytoin, and warfarin (during the protocol treatment)

3) Therapies that must be used with caution with TS-1 and UFT (radiotherapy, hyperthermia, immunotherapy, and hormones)

4) Use of investigational drugs

7.5.2. Permitted concomitant therapy

1) Concomitant use of medications for complications such as hypertension

2) Concomitant use of the following therapies not approved as antineoplastic therapy by the Japanese Ministry of Health, Labour and Welfare

a) Crude drugs such as hochu-ekkito, ninjin-yoeito, and juzen-taihoto

b) Dietary supplements such as agaricus, Phellinus linteus, Ganoderma lucidum, and shark cartilage

7.5.3. Recommended supportive therapies

The following supportive therapies will be recommended. However, not performing them will not be a protocol deviation.

1) WBC (leukocytes decreased): G-CSF

2) Hepatic disorders: liver protective agents, corticosteroid therapy, glucagon-insulin therapy, plasmapheresis, and intravenous amino acid solution

3) Serious renal disorders: Fluid management, electrolyte supplementation, renal dialysis

4) Serious enteritis or symptoms of dehydration: parenteral fluids, antidiarrheals, loperamide

5) Nausea, vomiting: antiemetics, minor tranquilizers

6) Stomatitis: allopurinol gargle, corticosteroids

7) Infections: antibiotics

7.6. Therapy following protocol treatment

This protocol does not specify any therapy after discontinuing the protocol treatment at relapse or developing secondary cancer. However, after discontinuation of the protocol treatment for other reasons and after completion of the protocol treatment, antineoplastics will not be administered until relapse or secondary cancer occurs or until 3 years have elapsed from enrollment of the last subject.

# Adverse Event Evaluation and Reporting

8.1. Definition of adverse event

An adverse event (AE) is any undesirable medical event occurring in a subject receiving the protocol treatment. An AE does not necessarily have to show a definite causal relationship with the protocol treatment. Thus, an adverse event is any undesirable or unintended sign (including abnormal clinical laboratory values), symptom, or disease occurring when the protocol treatment is received regardless of the causal relationship to the protocol treatment.

An AE is a serious adverse event (SAE) if it is any of the following.

1. Death

2. Life-threatening

3. Admission to a hospital or clinic or prolongation of admission that is necessary for treatment

4. Disability/incapacity

5. An event that may result in disability/incapacity

6. Any other serious event consistent with 1-5.

7. Congenital diseases or abnormalities in subsequent generations

8.2. Evaluation of adverse events

AEs observed during or within 30 days after completion of the protocol treatment will be evaluated according to the schedule in “9.2. Observation, test, and reporting schedule.” The maximum severity of each AE during each cycle will be recorded in the case report form.

The AEs observed will be evaluated according to the JCOG/JSCO Japanese-language version of the Common Terminology Criteria for Adverse Events (CTCAE) v.3.0. AEs will be graded according to the definitions (Grade 0-4) that most closely fit the event.

AEs for which a causal relationship to the drug cannot be ruled out will be considered adverse drug reactions.

# Observations, Tests, Reported Parameters, and Schedule

9.1. Observation and test parameters, and treatment information to be reported

9.1.1. Before enrollment (confirmation of eligibility) and baseline parameters

1) Information obtained before enrollment (entered in the enrollment form)

Basic patient information

Date of birth, age at consent (can be automatically calculated from date of birth and date of consent), sex, height at consent, weight at consent, body surface area at consent (can be automatically calculated from height and weight), date of written informed consent

Information on disease to be treated

Histological diagnosis: Squamous cell cancer

Primary site: Maxillary sinus, oral cavity, oropharynx, hypopharynx, larynx (except T3N0 of the glottis)

Baseline Stage: III, IVA, IVB

TNM staging: T(1, 2, 3, 4a, 4b), N(0, 1, 2, 3), M(0)

Curative therapy information

First curative therapy: surgery (date of surgery), radiotherapy (radiation duration and total dose), completion date of first curative therapy

Other concomitant therapy: Yes / No (If “Yes,” surgery, radiotherapy, chemotherapy [fluoropyrimidines, platinating agents, other ]),

Tumor eradication confirmation date, means of tumor eradication confirmation (diagnostic imaging, histopathology, cytology)

ECOG performance status scale: PS (0, 1)

Clinical laboratory tests (Test results obtained within 14 days before enrollment will be used.)

Test date

Hematology: WBC, Neut, PLT, Hb

Blood biochemistry: T.Bil, AST (GOT), ALT (GPT), Cr, albumin*, LDH*, ALP*, Na*,

K*, Ca*, CRP* Parameters marked with an asterisk will be entered into the baseline report.

Current medical history: Presence of infections, presence of gastrointestinal disorders, presence of interstitial pneumonia and/or pulmonary fibrosis, presence of diabetes mellitus, presence of heart disease, presence of hepatic disorders, presence of diarrhea (watery stools)

2) Baseline information (to be entered into the baseline report)

Recent cancer history: Presence of previous disease, diagnosis, year of diagnosis

Concurrent / previous disease besides cancer: Diabetes mellitus, hypertension, hyperlipidemia, coronary artery disease, stroke, other

Signs and symptoms (The highest grade in the 14 days before beginning the protocol treatment will be confirmed.):

Fatigue (malaise), weight loss, rash/desquamation, hyperpigmentation, anorexia, diarrhea, mucositis/stomatitis, nausea, vomiting, other (Remarkable signs and symptoms will be recorded.)

If the following parameters are measured, results obtained in the 30 days prior to enrollment will be used.

Tumor markers: Date performed, assay values (SCC or CYFRA)

Protocol treatment initiation date

Actual dose administered

9.1.2. During the study period

1) Testing dates

(1)TS-1-treated arm (experimental treatment arm)

The observations and tests listed below will be performed on the first dosing day (Day 1) and Day 8 of the first cycle and on the first day of the second and all subsequent cycles. The results will be entered in the case report forms via the web at the fourth and eighth cycles and at completion of treatment after 1 year.

(2)UFT-treated arm (control treatment arm)

The observations and tests listed below will be performed on the first dosing day (Day 1), Day 8, and Day 29 during the first 4 weeks and then every 4 weeks, starting at Week 5. The results will be entered in the case report forms via the web at 3, 6, and 12 months (completion of treatment).

2) Observations and tests: The following tests will be performed on the prescribed testing days.

Note that only the underlined data below will be reported via the web.

ECOG performance status scale: PS

Test parameters related to the study drugs

Compliance*: Compliance will be confirmed based on the medication record and scored on the following scale. The investigator will score compliance on the following 4-grade scale every 4 cycles for TS-1 and every 3 month for UFT.

*The percentage of drug taken during the dosing period will be calculated. Note that the number of capsules not taken during the rest period will not be included in the calculation.

(1) Took 100% (2) Took ≥ 75% and < 100%, (3) Took ≥ 50% and < 75%, (4) Took < 50%

Rest: presence of rest, reason for rest,

duration of rest (rest initiation date and treatment resumption date)

Dose reduction: presence of dose reduction, reason for dose reduction, daily dose after reduction

Discontinuation: presence of discontinuation, discontinuation date, reason for discontinuation

Submission of follow-up data after withdrawal of consent: possible, not possible

Clinical laboratory tests

Hematology: WBC, Neut, PLT, Hb

Blood biochemistry: T.Bil, AST, ALT, Cr, LDH*, ALP*, CRP*, Na*, K*, Cl*, Ca*

*Will be done when possible

(Only WBC, Neut, PLT, Hb, T.Bil, AST, ALT, and Cr will be entered in the record; the presence of adverse events in each test and the highest grade will be entered.)

Tumor markers: SCC or CYFRA

(Will be measured if necessary. Results will be entered via the web if measured within 30 days before or after discontinuation or completion of protocol treatment.)

Adverse events

Symptom terminology: Fatigue (malaise), weight loss, rash/desquamation, hyperpigmentation, anorexia, diarrhea, mucositis/stomatitis, nausea, vomiting, other (Remarkable events will be recorded.)

Grade: The highest grade during each case report form preparation period will be recorded.

Causal relationship: Yes, No

Confirmation of relapse and secondary cancer

Diagnostic imaging or pathological tests will be performed to confirm the presence of relapse or secondary cancer every 3-6 months after initiation of the protocol treatment. However, appropriate tests will be performed whenever relapse or secondary cancer is suspected from clinical symptoms or the like.

Presence of relapse (If present, the following items will be entered in the outcome report.)

Relapse site: local, distant (brain, bone, lung, liver, lymph node, adrenal gland, skin, other)

Confirmation method and date confirmation performed: simple chest radiography, ultrasonography, CT, MRI, PET, histopathology, cytology, other

Confirmation date (If diagnosed by multiple diagnostic methods, the date the earliest test was performed.)

Presence of secondary cancer (If present, the following items will be entered in the outcome report.)

Site of occurrence of secondary cancer (head and neck, lung, esophagus, stomach, other)

Diagnostic method and date diagnosis performed: simple chest radiography, ultrasonography, CT, MRI, PET, histopathology, cytology, other

Date of diagnosis (If diagnosed by multiple diagnostic methods, the date the earliest test was performed.)

*The presence of relapse and secondary cancer will be confirmed in accordance with “Definition of relapse and secondary cancer” in 11.1.

9.1.3. At follow-up

1) Testing dates

The observations and tests listed below will be performed every 3-6 months, counting from the first day of the protocol treatment. Follow-up will be performed for 3 years after the enrollment of the last subject. Entries in case report forms will be made once annually until Year 3, after which all tests and observations will be performed 3 years after the enrollment of the last subject.

2) Observations and tests

Outcome

Confirmation of outcome: Survival (date of last confirmation of survival)

Death (Cause of death: primary disease, other cancer, other, date of death)

Lost to follow-up (date of last confirmation of survival)

Method of confirmation: clinic appointment, other

*When survival status cannot be confirmed directly because the subject transferred to another hospital or the like, every effort will be made to obtain outcome information from the subject’s new hospital.

Confirmation of relapse and secondary cancer

Presence of relapse (If present, the following items will be entered in the outcome report.)

Relapse site: local, distant (brain, bone, lung, liver, lymph node, adrenal gland, skin, other)

Confirmation method and date confirmation performed: simple chest radiography, ultrasonography, CT, MRI, PET, histopathology, cytology, other

Confirmation date (If diagnosed by multiple diagnostic methods, the date the earliest test was performed.)

Presence of secondary cancer (If present, the following items will be entered in the outcome report.)

Site of occurrence of secondary cancer (head and neck, lung, esophagus, stomach, other)

Diagnostic method and date diagnosis performed: simple chest radiography, ultrasonography, CT, MRI, PET, histopathology, cytology, other

Date of diagnosis (If diagnosed by multiple diagnostic methods, the date the earliest test was performed.)

*Presence of relapse and secondary cancer will be confirmed in accordance with “Definition of relapse and secondary cancer” in 11.1.

Therapy following protocol treatment: Presence of therapy following protocol treatment

9.1.4. At occurrence of serious adverse events

The reporting technique in “8. Adverse Event Evaluation and Reporting” will be used.

9.2. Observation, test, and reporting schedule

∙Observations and tests (there will be a testing window of ± 14 days starting at Treatment Week 4 and ± 28 days during follow-up.)

| Time performed  Test | Enrollment | Treatment period | | | | | | | Discontinuation | Treatment completion | Follow-up † | Completion of follow-up |
| --- | --- | --- | --- | --- | --- | --- | --- | --- | --- | --- | --- | --- |
|  |  | TS-1-treated arm | | | UFT-treated arm | | | |  |  |  |  |
|  |  | 1st cycle | | Beginning of each subsequent cycle | Day 1 | Day 8 | Day  29 | Every 4 weeks thereafter |  | 1 year after starting protocol treatment | Every 3-6 months | 3 years after enrollment of last subject |
|  |  | Day 1 | Day 8 |  |  |  |  |  |  |  |  |  |
| Basic information / background | ● |  |  |  |  |  |  |  |  |  |  |  |
| PS | ● | ● | ● | ● | ● | ● | ● | ● | ● | ● |  |  |
| Compliance |  | ● | ● | ● | ● | ● | ● | ● | ● | ● |  |  |
| Clinical laboratory tests | ● | ● | ● | ●* | ● | ● | ● | ● | ● | ● |  |  |
| Signs and symptoms, adverse events | ● | ● | ● | ● | ● | ● | ● | ● | ● | ● |  |  |
| Tumor markers | ○ | ○ | ○ | ○ | ○ | ○ | ○ | ○ | ○ | ○ | ○ | ○ |
| Imaging or pathological tests | ○* (Every 3-6 months) | | | | | | | | | | | |
| Confirmation of relapse and secondary cancer | Confirmed as appropriate based on clinical symptoms, etc. | | | | | | | | | | | |
| Presence of therapy following protocol treatment |  |  |  |  |  |  |  |  |  | ● | ● | ● |
| Confirmation of outcome |  |  |  |  |  |  |  |  | ● | ● | ● | ● |

●: Must be done. ○: To be done when possible

●*: To ensure patient safety, it is desirable to perform clinical laboratory tests at times besides the beginning of each cycle.

○*: Diagnostic imaging or pathological tests will be performed every 3-6 months to confirm the presence of relapse or secondary cancer. However, appropriate tests will also be performed whenever relapse or secondary cancer is suspected.

†: Will be performed every 3-6 months, counting from the protocol treatment initiation day.

⋅ Reporting When reporting to the Data Center, data will be input according to the following schedule.

| Reporting time  Input sheet | Enrollment | Immediately after treatment initiation | Treatment period | | | | | | Follow-up |
| --- | --- | --- | --- | --- | --- | --- | --- | --- | --- |
|  |  |  | TS-1-treated arm | | | UFT-treated arm | | |  |
|  |  |  | 4th cycle  (12 weeks) | 8th cycle  (24 weeks) | Completion of protocol treatment  (1 year) | 3 months | 6 months | Completion of protocol treatment  (1 year) | Discontinuation / completion to 3 years after the enrollment of the last subject  (2 years, 3 years, and 3 years after the enrollment of the last subject) |
| Subject enrollment form | ● |  |  |  |  |  |  |  |  |
| Baseline report |  | ● |  |  |  |  |  |  |  |
| Treatment report |  |  | ● | ● | ● | ● | ● | ● |  |
| Outcome report |  |  |  |  | ● |  |  | ● | ● |

*All observations and tests are scheduled to be performed at one time 5 years after enrollment of the last subject.

# Target sample size and study period

10.1. Target sample size

The target sample size is 500 subjects (250 in the TS-1 arm and 250 in the UFT arm).

10.2. Study period

The enrollment and follow-up periods are as follows.

Enrollment period: 3 years (April 2006 to March 2009)

Follow-up period: 3 years after enrollment of the last subject (March 2012)

To study survival, all observations and tests are scheduled to be performed at one time 5 years after enrollment of the last subject (March 2014).

# Definitions of Endpoints

Events defining the endpoints are presented in the following table.

| Endpoint | Event (whichever is earlier) | | |
| --- | --- | --- | --- |
| Overall survival (OS) | Death from any cause | - | - |
| Relapse-free survival (RFS) | Death from any cause | Relapse | - |
| Disease-free survival (DFS) | Death from any cause | Relapse | Secondary cancer |

11.1. Primary endpoint

Disease-free survival (DFS)

The period from the enrollment date to earliest date among the date that relapse was diagnosed, date that secondary cancer was diagnosed, and date of death from any cause. For survivors not diagnosed with relapse or secondary cancer, DFS will be cut off at the last survival confirmation date.

Definition of relapse and secondary cancer

∙Definition of relapse and relapse confirmation date

“Relapse” will be diagnosed when any of the following criteria apply.

(1) Findings on diagnostic imaging (simple chest radiography, ultrasonography, CT, MRI, PET, etc.) that are not secondary cancer will be considered “relapse.” When relapse is diagnosed by multiple diagnostic methods, the date of the earliest test will be considered the “relapse confirmation date.”

(2) When relapse is diagnosed by pathological tests (cytology, biopsy), the date of the earliest test will be considered the “relapse confirmation date.”

(3) An increase in tumor markers will not be considered “relapse.”

∙Definition of secondary cancer (double cancer, multiple cancer) and secondary cancer confirmation date

(1) Definition of secondary cancer

Findings satisfying all of the criteria below from Warren and Gates Diagnostic Criteria 1) will be considered “secondary cancer.”

(1) Each of the tumors exhibits clear malignancy.

(2) Each of the tumors is separate and distinct.

(3) One tumor is not a metastasis of the other.

(2) Definition of secondary cancer confirmation date

(1) When there are findings on diagnostic imaging (simple chest radiography, ultrasonography, CT, MRI, PET, etc.), the date of the imaging will be considered the “confirmation date” and when secondary cancer is diagnosed by multiple diagnostic methods, the date of the earliest test will be considered the “secondary cancer confirmation date.”

(2) When secondary cancer is diagnosed by pathological tests (cytology, biopsy), the date of the earliest test will be considered the “confirmation date.”

11.2. Secondary endpoints

11.2.1. Relapse-free survival (RFS)

The period from the enrollment date to the earlier of the date that relapse was diagnosed or the date of death from any cause. For survivors not diagnosed with relapse, RFS will be cut off at the last survival confirmation date.

11.2.2. Overall survival (OS)

The period from the enrollment date to the date of death for any reason. OS will be cut off at the last survival confirmation date for subjects still alive at that time. For subjects lost to follow-up, OS will be cut off at the last survival confirmation date before they were lost to follow-up.

11.2.3. Adverse events

AEs will be graded according to the JCOG/JSCO Japanese-language version of CTCAE v.3.0.

# Statistical Considerations

12.1. Rationale for target sample size

The results of large-scale comparative studies in Western countries were used as the rationale for the disease-free survival rate in the control arm. This was done because there are no reports of Japanese studies in which UFT treatment was performed on control subjects as in the present study that could be evaluated.

The 3-year disease-free survival rate among subjects that had undergone curative therapy for Stage III, IVA, and IVB squamous cell cancers of the head and neck was approximately 35-45% in subjects that received radiotherapy postoperatively and approximately 40-59% in subjects that received combination radiotherapy and chemotherapy postoperatively.

From these results, it was hypothesized that the 3-year disease-free survival rate would be 50% in the control (UFT) arm. Thus, 216 subjects per arm would be required to have a hazard ratio of 0.7 (3-year disease-free survival rate: 61.6%) for the test (TS-1) arm versus the control arm with an enrollment period of 3 years, follow-up period of 3 years, two-sided α of 0.05, and statistical power of 80%. A sample size of 250 subjects per arm (500 in both arms) was selected because a power of test of at least 80% could be guaranteed with 250 subjects per arm.

12.2. Analysis set

The analysis set will consist of the group of all eligible subjects receiving part or all of the protocol treatment.

12.3. Analysis items and methods

12.3.1. Disease-free survival, overall survival, and relapse-free survival

Disease-free survival, overall survival, and relapse-free survival curves will be estimated by the Kaplan-Meier method. The stratified log-rank test, stratified by randomization adjustment factors, except for study site, will be used for between-group comparisons. To estimate the therapeutic effect, the Cox proportional hazards model will be used to find the hazards ratio of the therapeutic effects and its 95% confidence interval.

12.3.2. Adverse events

Adverse events will be tabulated by treatment arm and type, and Fisher’s exact test will be used to compare AE incidences.

12.4. Interim analysis

An interim analysis will be performed 1 year after completion of enrollment. The statistical significance of the inter-arm difference in overall survival will be studied, and an α consumption function will be used to make adjustments for the multiplicity of tests (the tests at the interim and final analysis) in order to maintain an α error of 5% for the study overall. An α consumption function of the O’Brien & Fleming type will be used.

# Independent Data Monitoring Committee

The independent data monitoring committee will evaluate the reports listed below at the request of the lead investigator and will provide recommendations to the lead investigator on protocol amendments (refer to “16. Protocol Amendments”) or early termination of the study (refer to “16.2 Early Termination of the Study”).

1) Study progress reports and study monitoring reports from the lead investigator (annually)

2) The interim analysis report from the statistician (1 year after enrollment of the last subject)

3) Serious adverse event reports sent from the lead investigator (as needed)

4) Relevant reports not from this study such as academic articles and presentations (as needed)

# Ethics-related items

14.1. Guideline compliance

All parties involved in this study will act in accordance with the Declaration of Helsinki of the World Medical Association and the Ethical Guidelines for Clinical Studies.

14.2. Preparation and revision of informed consent form and written information for subjects

The investigator will prepare the informed consent form and written information for subjects as well as the consent withdrawal form. Note that he may simply revise existing samples of informed consent forms and written information for subjects as well as consent withdrawal forms prepared by the lead investigator. The prepared informed consent form and written information for subjects will be submitted to the ethics committee at the investigator’s medical institution and the committee’s approval will be obtained prior to starting the study.

The written information for subjects must contain at least the following items. However, the written information must not be written so as to intentionally influence a subject to participate in the study.

1) That the study involves research

2) The study objectives

3) Study methods

4) The subject’s scheduled duration of participation in the study

5) The number of subjects scheduled to participate in the study

6) Anticipated clinical benefits and risks or inconvenience

7) When the subjects are patients, the availability of other treatment methods for the patient’s disease and the major anticipated benefits and risks of those treatments

8) Compensation and treatment that subjects can receive for physical injuries related to the study

9) That participation in the study is voluntary on the part of the subject and that the subject or his or her legal guardian can refuse to participate in the study or withdraw consent to participate in the study at any time. That the subject will not be treated unfairly as a result of refusing to participate or withdrawing consent, nor will the subject lose any benefits to which he or she is entitled as a result of not participating in the study.

10) That any information obtained that could affect willingness of the subject or his/her legal guardian to continue participating in the study will be promptly reported to the subject or the legal guardian

11) Conditions or reasons for discontinuation from the study

12) That persons engaged in monitoring or auditing, ethics committee members, and regulatory authorities will have access to original medical records. That the subject’s confidentiality will be protected as such times. That the subject or his/her legal guardian allows such access by writing his or her name and affixing his or her seal or by signing the informed consent form.

13) That the subject’s confidentiality will be protected if the results of the study are published

14) A description of any expenses to be borne by the subject

15) The name, title, and contact information of the investigator or subinvestigator

16) A point of contact at the study site where the subject should make inquiries or which the subject should contact if he or she desires further information regarding the study or subject’s rights or if a health injury occurs

17) The subject’s obligations in the study

18) That patent rights or the like may be arise from the results of this clinical study and ownership of any such patent rights, etc., if they arise

19) Funding sources, potential conflicts of interest, affiliations of researchers and others involved in the clinical study

20) The preparation date and version of the informed consent form

The formatted informed consent form must contain the following items.

1) The name of the clinical study

2) The preparation date and version of the written information for subjects

3) A section for the date that the study was explained and the name/seal or signature of the (sub)investigator

4) A section for the consent date and the name/seal or signature of the subject

5) A statement to the effect that the subject understands the explanation of the study and consents to participate in it

6) The name of the study site

The consent withdrawal form must contain the following items.

1) The name of the clinical study

2) A section for the name/seal or signature of the (sub)investigator

3) A section for the consent withdrawal date and the name/seal or signature of the subject

4) A statement to the effect that the subject is withdrawing consent to participate in the study

5) The name of the study site

If the investigator obtains any new information related to the subjects’ consent after study initiation and determines that it is necessary to revise the informed consent form and written information for subjects, the investigator will revise those forms. New information that affects subjects’ consent means, for example, new information on adverse events related to the test treatment method or information related to the development of new therapeutic methods for the disease treated. If it is determined that the revisions are significant, they will be submitted to the ethics committee of the investigator’s medical institution and the committee’s approval will be obtained.

14.3. Informed consent

Before the subject participates in the study, the (sub)investigator will thoroughly explain the study, using the written information for subjects, and obtain voluntary written consent to participate in the study from the subject personally.

When obtaining written consent, the (sub)investigator who explained the study and the subject, after the subject completely understanding the written information for subjects, will each enter the dates, print names/seal or sign the informed consent form.

The (sub)investigator will give the subject a copy of the sealed (with written names) or signed informed consent form and the written information for subjects and attach the original informed consent form to the subject’s medical records which will be stored at the medical institution.

When there is a major revision to the written information for subjects, the (sub)investigator will use the revised written information for subjects to give another explanation of the study to subjects participating in the study and obtain the voluntary written consent of each subject personally to continue participation in the study.

When a subject requests to withdraw consent while participating in the study, the (sub)investigator and the subject will each enter the date, print names/seal or sign a statement to that effect (a consent withdrawal form). A copy of the consent withdrawal form will be given to the subject, and the original will be attached to the medical records and stored at the medical institution.

14.4. Protection of personal information

Persons involved in the study will strictly protect the personal information of subjects in accordance with based on the Personal Information Protection Law.

When providing subject enrollment forms, case report forms, and the like to parties outside the medical institution, the (sub)investigator will assign and use new subject identification codes for performing linkable anonymization. Persons outside the medical institution will not record information by which subjects can be identified (e.g., name, address, and phone number).

When the Data Center makes inquiries to the medical institution, subjects will be identified by the subject identification codes controlled by the (sub)investigator or the enrollment number issued by the Data Center.

When accessing raw source materials directly, monitoring, audit, regulatory agency, and other personnel will not disclose the information thus obtained to outside parties.

When data obtained in the study is published, the lead investigator, etc., will take thorough measures to ensure that the subjects cannot be identified.

# Financial Burden for the Study

15.1. Funding sources and potential conflict of interest

The present clinical study received funding from Taiho Pharmaceutical Co., Ltd. and will be conducted as a clinical study of the Foundation for Biomedical Research and Innovation. The results of the clinical study will be published whether favorable or not.

15.2. Expenses related to the clinical study

This clinical study is being conducted within the scope of standard health insurance. The observations and tests performed and drugs used during the study period will be covered by the subjects’ health insurance.

15.3. Compensation for health injury

When there are adverse events caused by this clinical study and subjects experience health injury, the lead investigator, investigators, subinvestigators, and medical institutions will provide therapy and other suitable measures. However, the therapy and other services provided will be covered by health insurance and no other monetary compensation will be provided.

# Protocol Amendments

When it is necessary to amend the protocol, the lead investigator will obtain the approval of protocol committee members before making the amendment, and will report a description of the amendment and the reason for it to ethics committee of his research institution. When a protocol amendment is considered significant, it must also be reviewed again and approved by the ethics committee of the lead investigator’s research institution. It will be left to the discretion of the investigators at the study sites to determine of if another review is necessary.

If it is necessary to suspend enrollment while the protocol is being amended, the lead investigator will inform the following parties of that fact: the investigators, subinvestigators, independent data monitoring committee members, Data Center, and statistician.

After amending the protocol, the lead investigator will send the amended protocol or a description of the amendment to the investigators, subinvestigators, independent data monitoring committee members, Data Center, and statistician. The investigators will revise the written information for subjects, based on the protocol amendment. For amendment procedures, refer to “14.2. Preparation and revision of informed consent form and written information for subjects.”

# Completion and Early Termination of the Study

17.1. Completion of the study

The study will be completed when the follow-up period (refer to “10.2. Study period”) is completed and the database is locked. After the lead investigator is informed by the Data Center that the database has been locked, he will inform the investigators, statistician, and independent data monitoring committee members that the study has been completed. The investigators will report this information to the heads of the medical institutions and departments involved in the study at the medical institutions.

17.2. Early termination of the study

17.2.1. Rules for early termination of the study

The study will be terminated early in the following cases.

1) As a result of evaluating study progress reports from the lead investigator and study monitoring reports, it is concluded that completion of the study is compromised by slow enrollment, frequency of protocol deviations, or the like.

2) Interim analysis confirms the superiority of the test treatment’s efficacy.

3) Interim analysis confirms the inferiority of the test treatment’s efficacy.

4) Adverse events satisfying the following criteria are observed, and it is determined that there are problems with the safety of the protocol treatment.

∙ Protocol-treatment-related deaths 10 subjects

∙ Protocol-treatment-related serious adverse events, including deaths 25 subjects

∙ Protocol-treatment-related Grade 4 neutropenia 25 subjects

5) It is concluded that there are problems with the safety of the protocol treatment as a result of evaluating related information obtained from other studies, scholarly articles, academic conferences, or the like, or it is concluded that continuation of the study is no longer significant.

17.2.2. Procedure for determining to terminate the study early

The lead investigator must request that the independent data monitoring committee convene and accept recommendations on early termination of the study in accordance with “13. Independent Data Monitoring Committee.” Based on these recommendations, the lead investigator will conclude whether early termination is necessary according to the rules in the previous section. If the recommendations are not followed, the lead investigator will report the reason to the independent data monitoring committee.

If the lead investigator decides to terminate the study early, he will immediately inform the following parties of the reason and subsequent actions to be taken: the investigators, statistician, Data Center, and independent data monitoring committee members. After notification of this, the investigators will report that the study was terminated early and the reason for it to the subjects and immediately take the proper actions. In addition, the investigators will report this to the heads of the medical institutions and the departments involved in the study at the medical institutions.

# References

1) Laramore GE, Scott CB, al-Sarraf M, *et al* : Adjuvant chemotherapy for respectable squamous cell carcinomas of the head and neck: report on Intergroup Study 0034. *Int J Radiot Oncol Biol Phys* 23(4): 705-13, 1992

2) Bernier J, Domenge C, Ozsahin M, *et al* : Postoperative irradiation with or without Concomitant Chemotherapy for Locally Advanced Head and Neck Cancer. *N Engl J Med* 350(19):1945-1952, 2004

3) Cooper JS, Pajak TF, Forastiere AA, *et al* : Postoperative Concurrent Radiotherapy and Chemotherapy for High-Risk Squamous-Cell Carcinoma of the Head and Neck. *N Engl J Med* 350(19):1937-1944, 2004

4) Miyake H, Takeda C, Okuda M, *et al* : Adjuvant Therapy with Bestatin for Squamous Cell Carcinoma of the Head and Neck - a multicenter, randomized controlled study-[Article in Japanese]. *Otologia Fukuoka [Jibi to Rinsho]*, 30(6): 1142-51, 1984

5) Tsukuda M, Ogasawara H, Kaneko S, *et al* : Randomized comparison of the usefulness of UFT as adjuvant chemotherapy for head and neck cancer [Article in Japanese]. *Japanese Journal of Cancer and Chemotherapy* 21(8):1169-77, 1994

6) Inuyama Y, Kida A, Tsukuda M, Kohno N, Satake B; S-1 Cooperative Study Group (Head and Neck Cancer Working Group): Early phase II study of S-1 in patients with head and neck cancer [Article in Japanese]. *Japanese Journal of Cancer and Chemotherapy* 25(8): 1151-8, 1998

7) Inuyama Y, Kida A, Tsukuda M, Kohno N, Satake B; S-1 Cooperative Study Group (Head and Neck Cancer Working Group): Late phase II study of S-1 in patients with advanced head and neck cancer. [Article in Japanese]. *Japanese Journal of Cancer and Chemotherapy* 28(10):1381-90, 2001

8) Nagashima F, Ohtsu A, Yoshida S and Ito K: Japanese nationwide post-marketing survey of S-1 in patients with advanced gastric cancer. *Gastric Cancer* 8:6-11, 2005

9) Kimura Y, Kikkawa N, Iijima S, , *et al* : A new regimen for TS-1 therapy designed to minimize adverse reactions by introducing a one-week interval after each two-week dosing session [Article in Japanese]. *Japanese Journal of Cancer and Chemotherapy* 29(8): 1403-9, 2002

10) Tsukuda M, Kida A, Fujii M, *et al* : Randomized scheduling feasibility study of S-1 for adjuvant chemotherapy in advanced head and neck cancer. *Br J Cancer* 93:884-889, 2005

11) Kato H, Ichinose Y, Ohta M, *et al* : A Randomized Trial of Adjuvant Chemotherapy with Uracil-Tegafur for Adenocarcinoma of the Lung. *N E J Med* 350(17):1713-21, 2004

12) Akasu T, Moriya S, Yoshida S, *et al* : Adjuvant oral uracil and tegafur (UFT) improves survival after complete mesorectal excision (ME) for pathologic TNM stage III rectal cancer (RC): Results of the National Surgical Adjuvant Study (NSAS)-Colorectal Cancer (CC) 01 randomized trial. Proceedings of the 40^th^ ASCO Annual Meeting; 2004 June 6-8 ; New Orleans, USA. 2004. p251

13) Kinoshita T, Nakajima T, Ohashi Y, *et al* :Adjuvant chemotherapy with uracil-tegafur (UFT) for serosa negative advanced gastric cancer: Results of a randomized trial by national surgical adjuvant study of gastric cancer. Proceedings of the 41th ASCO Annual Meeting; 2005 May 14-16 ; Olando, USA. 2005. p313

References for the Section on Statistical Considerations

1) Warren S and Gates O: Multiple primary malignant tumors. A survey of literature and statistical study. Am. J. Cancer 16(6):1358-1414, 1932

2) Cooper JS, Pajak TF, Forastiere AA et al: Postoperative concurrent radiotherapy and chemotherapy for high-risk squamous-cell carcinoma of the head and neck. N Engl J Med 350(19):1937-1944, 2004

3) Bernier J, Domenge C, Ozsahin M et al: Postoperative irradiation with or without concomitant chemtoherapy for locally advanced head and neck cancer. N Engl J Med 350(19):1945-1952, 2004

4) Laramore GE, Scott CB, Al-Sarraf M et al: Adjuvant chemtoherapy for resectable squamous cell carcinoma of the head and neck: report on INTERGROUP study 0034. Int J Radiat Oncol Biol Phys 23: 705-713, 1992

5) Pocock SJ, Simon R.: Sequential treatment assignment with balancing for prognostic factors in the controlled clinical trial. Biometrics. 1975;31:103-115
