# Supplementary material for: Randomized Phase III Trial of Adjuvant Chemotherapy with S-1 after Curative Treatment in Patients with Squamous-Cell Carcinoma of the Head and Neck (ACTS-HNC)
Source: PLoS One. 2015 Feb 11;10(2):e0116965. doi: 10.1371/journal.pone.0116965 (PMC4324826; doi:10.1371/journal.pone.0116965)
Supplement: S1 List — (PDF) [file pone.0116965.s003.pdf]

(ACTS-HNC) List of the name of committee/institutional review boards

If there is a difference between the Japanese and English sentence, original Japanese takes precedence.

	institution (Japanese)	institution (English)	name of committee/institutional review boards (Japanese, original)	name of committee/institutional review boards (English, reference)	Approval number	statement indicating (Japanese, original)	statement indicating (English, reference)	Apporoved date (yyyy/mm/dd)
1	北海道大学病院	Hokkaido University Graduate School of Medicine	倫理審査委員会	Independent Ethics Committee	－	承認	Approval	2006/3/31
2	札幌医科大学大学院医学研究科	Sapporo Medical University	臨床研究審査委員会	IRB	1-18	承認	Approval	2006/6/30
3	旭川医科大学	Asahikawa Medical University	倫理委員会	Independent Ethics Committee	250	承認	Approval	2006/3/14
4	独立行政法人国立病院機構 北海道がんセンター	Hokkaido Cancer Center	治験審査委員会	IRB	60E01	了承	Approval	2006/4/13
5	福島県立医科大学	Fukushima Medical University	倫理審査委員会	Independent Ethics Committee	－	承認	Approval	2006/5/12
6	秋田大学医学部	Akita University Graduate School of Medicine and Faculty of Medicine	倫理審査委員会	Independent Ethics Committee	－	承認	Approval	2006/4/17
7	山形大学医学部 情報構造統御学講座	Yamagata University School of Medicine	審査委員会	Independent Ethics Committee	91	承認	Approval	2006/10/16
8	東北大学病院	Tohoku University School of Medicine	倫理委員会	Independent Ethics Committee	2006-108	承認	Approval	2006/6/19
9	弘前大学医学部附属病院	Hirosaki University School of Medicine and Hospital	倫理委員会	Independent Ethics Committee	－	承認	Approval	2006/4/28
10	岩手医科大学附属病院	Iwate Medical University Hospital	倫理委員会	Independent Ethics Committee	H18-23	承認	Approval	2006/7/6
11	新潟県立中央病院	Niigata Prefectural Central Hospital	治験審査委員会	IRB	－	修正の上で承認	Contingent Approval	2006/4/6
12	栃木県立がんセンター	Tochigi Cancer Center	臨床研究審査委員会	IRB	－	承認	Approval	2006/6/21
13	埼玉県立がんセンター	Saitama Cancer Center	臨床研究審査委員会	IRB	－	承認	Approval	2006/3/28
14	群馬大学医学部附属病院	Gunma University Hospital	臨床試験審査委員会	IRB	477	承認	Approval	2006/3/31
15	獨協医科大学病院	Dokkyo Medical University Hospital	生命倫理委員会	Independent Ethics Committee	1762	賛成	Approval	2006/3/24
16	獨協医科大学越谷病院	Dokkyo Medical University Koshigaya Hospital	治験審査委員会	IRB	－	承認	Approval	2006/4/24
17	自治医科大学医学部	Jichi Medical University	疫学研究倫理審査委員会	Independent Ethics Committee	臨06-29号	承認	Approval	2006/8/21
18	千葉大学大学院医学研究院	Graduate School of Medicine and School of Medicine, Chiba University	治験審査委員会	IRB	－	承認	Approval	2006/5/17
19	東京歯科大学市川総合病院	Tokyo Dental College Ichikawa General Hospital	倫理委員会	Independent Ethics Committee	89	承認	Approval	2006/3/2
20	慶應義塾大学医学部	Keio University	倫理委員会	Independent Ethics Committee	18-21(3)	承認	Approval	2006/10/11
21	東京医科歯科大学大学院医歯学総合研究科	Tokyo Medical and Dental University	IRB	IRB	－	承認	Approval	2006/7/24
22	癌研究会有明病院	Cancer Institute Hospital	治験・臨床研究倫理審査委員会	IRB	第2005-0066号	承認	Approval	2006/5/17
23	独立行政法人国立病院機構 東京医療センター	National Hospital Organization Tokyo Medical Center	倫理委員会	Independent Ethics Committee	－	承認	Approval	2006/5/24
24	日本医科大学	Nippon Medical School	倫理審査委員会	Independent Ethics Committee	－	承認	Approval	2006/5/12
25	順天堂大学医学部 附属順天堂医院	Juntendo University Hospital	病院倫理審査委員会	Independent Ethics Committee	112	条件付承認	Contingent Approval	2006/3/16
27	国立国際医療センター	National Center for Global Health and Medicine	受託研究審査委員会	IRB	H-039-06f	承認	Approval	2006/4/21
28	東京医科大学	Tokyo Medical University	医学倫理委員会	Independent Ethics Committee	655	承認	Approval	2006/5/31
29	東京都立駒込病院	Tokyo Metropolitan Komagome Hospital	受託研究審議委員会	IRB	－	承認	Approval	2006/7/19
30	杏林大学	Kyorin University	倫理委員会	Independent Ethics Committee	170	承認	Approval	2006/5/22
31	山梨大学大学院 医学工学総合研究部	University of Yamanashi Graduate School of Medical Science	倫理委員会	Independent Ethics Committee	259	承認	Approval	2006/4/27
32	東京医科大学 八王子医療センター	Tokyo Medical University Hachioji Medical Center	臨床研究倫理審査委員会	Independent Ethics Committee	－	修正の上で承認	Contingent Approval	2006/3/27
33	青梅市立総合病院	Ome Municipal General Hospital	治験審査委員会	IRB	－	承認	Approval	2006/4/18
34	横浜市立大学医学部	Yokohama City University School of Medicine	倫理委員会	Independent Ethics Committee	05-050	承認	Approval	2006/3/6
35	横浜市立大学附属 市民総合医療センター	Yokohama City University Medical Center	倫理委員会	Independent Ethics Committee	404	承認	Approval	2006/3/27
36	東海大学	Tokai University	倫理委員会	Independent Ethics Committee	臨審委(受)<br>第05-073号	承認	Approval	2006/3/27
37	北里大学医学部	Kitasato University	病院倫理委員会	Independent Ethics Committee	C倫06-236	承認	Approval	2006/6/21
38	神奈川県立がんセンター	Kanagawa Cancer Center	IRB	IRB	－	承認	Approval	2006/5/17
40	昭和大学藤ヶ丘病院	Showa University Fujigaoka Hospital	IRB	IRB	2006520	承認	Approval	2006/7/21
41	浜松医科大学	Hamamatsu University School of Medicine	倫理委員会	Independent Ethics Committee	第18-16号	承認	Approval	2006/6/28
42	静岡県立静岡がんセンター	Shizuoka Cancer Center	倫理審査委員会	Independent Ethics Committee	18-62-18-1-2	修正の上で承認	Contingent Approval	2007/2/28
43	信州大学医学部	Shinshu University	倫理委員会	Independent Ethics Committee	786	承認	Approval	2006/7/25
44	藤田保健衛生大学	Fujita Health University	疫学・臨床研究倫理審査委員会	Independent Ethics Committee	06-027	承認	Approval	2006/3/14
45	三重大学医学部附属病院	Mie University Hospital	医学部研究倫理審査委員会	Independent Ethics Committee	669	承認	Approval	2006/6/26
46	藤田保健衛生大学 坂文種報徳會病院	Fujita Health University Banbuntane Houtokukai Hospital	疫学・臨床研究倫理審査委員会	Independent Ethics Committee	06-057	承認	Approval	2006/6/21
47	愛知県がんセンター中央病院	Aichi Cancer Center Hospital	倫理審査委員会	Independent Ethics Committee	8-16	承認	Approval	2006/7/25
48	名古屋第一赤十字病院	Japanese Red Cross Nagoya Daiichi Hospital	治験審査委員会	IRB	－	承認	Approval	2006/6/1
49	岐阜大学医学部附属病院	Gifu University Hospital	医学研究等倫理審査委員会	Independent Ethics Committee	18-21	承認	Approval	2006/5/10
50	市立四日市病院	Yokkaichi Municipal Hospital	倫理審査委員会	Independent Ethics Committee	－	承認	Approval	2006/4/14
51	金沢大学医学部附属病院	Kanazawa University School of Medicine	倫理審査委員会	Independent Ethics Committee	5334	承認	Approval	2006/5/12
52	金沢医科大学	Kanazawa Medical University	IRB	IRB	73	許可	Approval	2006/9/26
53	福井大学医学部附属病院	University of Fukui	医薬品等臨床研究審査委員会	IRB	2006801	承認	Approval	2006/4/7
54	京都府立医科大学	Kyoto Prefectural University of Medicine	医学倫理審査委員会	Independent Ethics Committee	C-166	許可	Approval	2006/8/15
55	精光会　草津総合病院	Kusatsu General Hospital	倫理審査委員会	Independent Ethics Committee	－	承認	Approval	2007/1/23
56	大阪市立大学大学院 医学研究科	Osaka City University Graduate School of Medicine	倫理委員会	Independent Ethics Committee	受研　第2809号	承認	Approval	2006/3/27
57	大阪府立成人病センター	Osaka Medical Center for Cancer and Cardiovascular Diseases	倫理審査委員会	Independent Ethics Committee	－	承認	Approval	2006/4/5
58	奈良県立医科大学	Nara Medical University	倫理審査委員会	Independent Ethics Committee	－	承認	Approval	2006/10/1
59	関西医科大学附属枚方病院	Kansai Medical University Hirakata Hospital	治験審査委員会	IRB	第H060503号	許可	Approval	2006/5/11
60	和歌山県立医科大学	Wakayama Medical University	倫理委員会	Independent Ethics Committee	392	承認	Approval	2006/5/1
61	大阪府立 急性期総合医療センター	Osaka General Medical Center	治験・臨床研究審査委員会	IRB	－	承認	Approval	2006/3/17
62	大阪医科大学	Osaka Medical College Hospital	倫理委員会	Independent Ethics Committee	0365	承認	Approval	2006/5/1
63	近畿大学医学部	Kinki University Faculty of Medicine	倫理委員会	Independent Ethics Committee	18-02	承認	Approval	2006/4/28
64	天理よろづ相談所病院	Tenri Hospital	倫理委員会	Independent Ethics Committee	187	承認	Approval	2006/3/24
65	神戸大学医学部附属病院	Kobe University	IRB	IRB	437	承認	Approval	2006/7/12
66	兵庫医科大学	Hyogo College of Medicine	倫理委員会	Independent Ethics Committee	351	承認	Approval	2006/6/16
68	独立行政法人国立病院機構 姫路医療センター	National Hospital Organization Himeji Medical Center	倫理審査委員会	Independent Ethics Committee	－	承認	Approval	2006/8/4
69	広島大学病院	Hiroshima University Hospital	倫理委員会	Independent Ethics Committee	629	承認	Approval	2006/8/31
70	鳥取大学医学部	Tottori University Faculty of Medicine	IRB	IRB	653	承認	Approval	2006/4/11

71	島根大学医学部附属病院	Shimane University Faculty of Medicine	臨床研究審査部会	IRB	18-自-03	承認	Approval	2006/4/18
72	山口大学医学部	Yamaguchi University School of Medicine	倫理審査委員会	Independent Ethics Committee	−	承認	Approval	2006/4/26
73	川崎医科大学	Kawasaki Medical School	受託研究審査委員会	IRB	180361	承認	Approval	2006/3/29
74	香川大学医学部	Kagawa University faculty of Medicine	IRB	IRB		承認	Approval	2006/5/19
75	愛媛大学医学部附属病院	Ehime University	IRB	IRB	−	承認	Approval	2006/3/27
76	独立行政法人国立病院機構 九州がんセンター	National Hospital Organization Kyushu Cancer Center	倫理委員会	Independent Ethics Committee	2006-27	承認	Approval	2006/7/25
77	久留米大学病院	Kurume University School of Medicine	倫理委員会	Independent Ethics Committee	−	承認	Approval	2006/7/5
78	九州大学病院	Kyushu University Hospital	臨床試験審査委員会	IRB	18017	承認	Approval	2006/10/13
79	佐賀大学医学部	Saga Medical School Faculty of Medicine,Saga University	臨床研究倫理審査委員会	Independent Ethics Committee	2006-04-05	承認	Approval	2006/7/3
80	大分大学医学部	Oita University	倫理委員会	Independent Ethics Committee	19-26	承認	Approval	2006/7/20
81	宮崎大学医学部附属病院	University of Miyazaki	倫理委員会	Independent Ethics Committee	243	承認	Approval	2006/6/13
82	独立行政法人国立病院機構 九州医療センター	National Kyushu Medical Center	倫理審査委員会	Independent Ethics Committee	06H14	承認	Approval	2006/7/19
83	福岡大学病院	Fukuoka University	臨床研究審査委員会	IRB	6-5	承認	Approval	2006/4/26
84	鹿児島大学医学部・歯学部附属病院	Kagoshima University Medical And Dental Hospital	臨床研究倫理委員会	Independent Ethics Committee	18-4	承認	Approval	2006/4/7
85	熊本大学医学部附属病院	Kumamoto University	倫理審査委員会	Independent Ethics Committee	−	承認	Approval	2006/4/27
86	鹿児島市立病院	Kagoshima City Hospital	臨床試験審査委員会	IRB	−	承認	Approval	2006/4/20
87	琉球大学	University of the Ryukyus	倫理審査委員会	Independent Ethics Committee	−	承認	Approval	2006/9/20
88	埼玉医科大学　国際医療センター	Saitama Medical University International Medical Center	IRB	IRB	06-003	承認	Approval	2006/12/7
89	聖隷浜松病院	Seirei Hamamatsu General Hospital	臨床研究審査委員会	IRB	269	承認	Approval	2006/3/16
90	独立行政法人労働者健康福祉機構 横浜労災病院	Yokohama Rosai Hospital	倫理審査委員会	Independent Ethics Committee	−	承認	Approval	2006/3/6
91	済生会宇都宮病院	Saiseikai Utsunomiya Hospital	倫理委員会	Independent Ethics Committee	−	承認	Approval	2006/2/10
92	大阪府済生会中津病院	Osaka Saiseikai Nakatsu Hospital	倫理委員会	Independent Ethics Committee	06-40	承認	Approval	2006/9/5
93	独立行政法人国立病院機構 熊本医療センター	National Hospital Organization Kumamoto Medical Center	倫理審査委員会	Independent Ethics Committee	122	承認	Approval	2006/10/3
94	神戸市立医療センター中央市民病院	Kobe City Medical Center General Hospital	臨床研究審査委員会	IRB	3	承認	Approval	2006/10/16
95	兵庫県立がんセンター	Hyogo Cancer Center	共同研究審査委員会	IRB	−	承認	Approval	2007/6/20
96	新潟県立がんセンター新潟病院	Niigata Cancer Center Hospital	倫理審査委員会	Independent Ethics Committee	31	承認	Approval	2007/10/9
97	大分県立病院	Oita Prefectural Hospital	倫理審査委員会	Independent Ethics Committee	19-26	承認	Approval	2008/3/7
98	愛知医科大学	Aichi Medical University	倫理審査委員会	Independent Ethics Committee	526	承認	Approval	2008/3/3
