# Supplementary material for: Randomized Phase III Trial of Adjuvant Chemotherapy with S-1 after Curative Treatment in Patients with Squamous-Cell Carcinoma of the Head and Neck (ACTS-HNC)
Source: PLoS One. 2015 Feb 11;10(2):e0116965. doi: 10.1371/journal.pone.0116965 (PMC4324826; doi:10.1371/journal.pone.0116965)
Supplement: S2 Table — (DOCX) [file pone.0116965.s005.docx]

|  | | Treatment resumption criterion |
| --- | --- | --- |
| Hematologic toxicities | Leukocytes | ≥ 3,000 mm^-3^ |
|  | Neutrophils | ≥ 1,500 mm^-3^ |
|  | Platelets | ≥ 100,000 mm^-3^ |
|  | Hemoglobin | ≥ 9.0 g dL^-1^ |
| Non-hematologic toxicities | Total bilirubin | < 1.5 mg dL^-1^ |
|  | AST, ALT | < 100 IU L^-1^ |
|  | Creatinine | < 1.2 mg dL^-1^ |
|  | Other adverse events | ≤ Grade 1 |

S2 table. Criteria for treatment resumption
