# Supplementary material for: Randomized Phase III Trial of Adjuvant Chemotherapy with S-1 after Curative Treatment in Patients with Squamous-Cell Carcinoma of the Head and Neck (ACTS-HNC)
Source: PLoS One. 2015 Feb 11;10(2):e0116965. doi: 10.1371/journal.pone.0116965 (PMC4324826; doi:10.1371/journal.pone.0116965)
Supplement: S3 Table — (DOCX) [file pone.0116965.s006.docx]

|  | | Criterion for dose reduction |
| --- | --- | --- |
| Hematologic toxicities | Leukocytes | < 2,000 mm^-3^ (≥ Grade 3) |
|  | Neutrophils | < 1,000 mm^-3^ (≥ Grade 3) |
|  | Platelets | < 50,000 mm^-3^ (≥ Grade 3) |
|  | Hemoglobin | < 8.0 g dL^-1^ (≥ Grade 3) |
| Non-hematologic toxicities | Total bilirubin | ≥ 3 mg dL^-1^ |
|  | AST, ALT | ≥ 150 IU L^-1^ |
|  | Creatinine | ≥ 1.2 mg dL^-1^ |
|  | Other adverse events | ≥ Grade 3 |
| In addition, the dose was reduced if the attending physician judged that dose reduction was needed. | | |

S3 table. Criteria for dose reduction
