# Supplementary material for: Randomized Phase III Trial of Adjuvant Chemotherapy with S-1 after Curative Treatment in Patients with Squamous-Cell Carcinoma of the Head and Neck (ACTS-HNC)
Source: PLoS One. 2015 Feb 11;10(2):e0116965. doi: 10.1371/journal.pone.0116965 (PMC4324826; doi:10.1371/journal.pone.0116965)
Supplement: S4 Table — (DOCX) [file pone.0116965.s007.docx]

| Body-surface area | Starting dose  (tegafur equivalent) | UFT dose level | |
| --- | --- | --- | --- |
|  |  | 1st dose reduction | 2nd dose reduction |
| < 1.5 m^2^ | 300 mg day^-1^ | Discontinuation | - |
| ≥ 1.5 m^2^ | 400 mg day^-1^ | 300 mg day^-1^ | Discontinuation |

S4 Table. Levels for UFT dose reduction
