# Supplementary material for: Randomized Phase III Trial of Adjuvant Chemotherapy with S-1 after Curative Treatment in Patients with Squamous-Cell Carcinoma of the Head and Neck (ACTS-HNC)
Source: PLoS One. 2015 Feb 11;10(2):e0116965. doi: 10.1371/journal.pone.0116965 (PMC4324826; doi:10.1371/journal.pone.0116965)
Supplement: S5 Table — (DOCX) [file pone.0116965.s008.docx]

| Body surface area | Starting dose  (tegafur equivalent) | TS-1 dose level | | |
| --- | --- | --- | --- | --- |
|  |  | 1st dose reduction | 2nd dose reduction | 3rd dose reduction |
| < 1.25 m^2^ | 80 mg day^-1^ | Discontinuation | - | - |
| ≥ 1.25 m^2^ to < 1.5 m^2^ | 100 mg day^-1^ | 80 mg day^-1^ | Discontinuation | - |
| ≥ 1.5 m^2^ | 120 mg day^-1^ | 100 mg day^-1^ | 80 mg day^-1^ | Discontinuation |

S5 Table. Levels for S-1 dose reduction
